# *Dictyostelium discoideum* cells retain nutrients when the cells are about to outgrow their food source

**DOI:** 10.1242/jcs.260107

**Published:** 2022-09-21

**Authors:** Ramesh Rijal, Sara A. Kirolos, Ryan J. Rahman, Richard H. Gomer

**Affiliations:** Department of Biology, Texas A&M University, College Station, TX 77843-3474, USA

**Keywords:** Polyphosphate, Macropinocytosis, Exocytosis, Membrane recycling, Membrane fluidity, *Dictyostelium*

## Abstract

*Dictyostelium discoideum* is a unicellular eukaryote that eats bacteria, and eventually outgrows the bacteria. *D. discoideum* cells accumulate extracellular polyphosphate (polyP), and the polyP concentration increases as the local cell density increases. At high cell densities, the correspondingly high extracellular polyP concentrations allow cells to sense that they are about to outgrow their food supply and starve, causing the *D. discoideum* cells to inhibit their proliferation. In this report, we show that high extracellular polyP inhibits exocytosis of undigested or partially digested nutrients. PolyP decreases plasma membrane recycling and apparent cell membrane fluidity, and this requires the G protein-coupled polyP receptor GrlD, the polyphosphate kinase Ppk1 and the inositol hexakisphosphate kinase I6kA. PolyP alters protein contents in detergent-insoluble crude cytoskeletons, but does not significantly affect random cell motility, cell speed or F-actin levels. Together, these data suggest that *D. discoideum* cells use polyP as a signal to sense their local cell density and reduce cell membrane fluidity and membrane recycling, perhaps as a mechanism to retain ingested food when the cells are about to starve.

This article has an associated First Person interview with the first author of the paper.

## Teaser

**Summary:** *Dictyostelium discoideum* cells at high cell densities use extracellular polyphosphate to alter membrane properties and inhibit exocytosis of undigested or partially digested nutrients using a signal transduction pathway.

## INTRODUCTION

Some animals use seasonal cues to anticipate starvation during winter, and store food as body fat for hibernation (Florant and Healy, 2012). A similar process occurs in the unicellular eukaryote *Dictyostelium discoideum*, which uses polyphosphate (polyP; linear chains of phosphate groups) as a secreted signal to monitor the local cell density, and when there is a high density of cells that will soon outgrow the local food supply, the cells stop proliferating but continue to grow (accumulate mass and protein) in anticipation of starvation (Soll et al., 1976; Yarger et al., 1974). PolyP inhibits proliferation by inhibiting cytokinesis, but has relatively little effect on growth (Suess and Gomer, 2016). In yeast, mutations that inhibit proliferation but do not inhibit growth exist (Johnston et al., 1977), and conversely some mouse embryos during early development have cell proliferation without growth (Johnston et al., 1977; Nasmyth, 1996; Su and O'Farrell, 1998). However, much remains to be understood about how cells separately regulate growth and proliferation.

*D. discoideum* is a soil dwelling amoeba that shares common ancestors with plants and animals (Baldauf and Doolittle, 1997). During growth, the cells feed on bacteria and divide. When starved, *D. discoideum* cells aggregate to form fruiting bodies consisting of a mass of spore cells held above the ground by a column of stalk cells. Dispersal of spores to a moist environment causes the spores to hatch and germinate into amoeba (Kessin, 2001; Loomis, 2014; Schaap, 2011). For nutrient reserves, *D. discoideum* cells store lipid in the form of lipid droplets (Kornke and Maniak, 2017), and glycogen, which is broken down during development (Garrod and Ashworth, 1972; Harris and Rutherford, 1976).

PolyP is present in many cell types (Rao et al., 2009). In prokaryotes, polyP is synthesized from ATP by polyphosphate kinase 1 (Ppk1) (Ahn and Kornberg, 1990; Brown and Kornberg, 2008), and is involved in stress responses, virulence, quorum sensing and biofilm formation (Rao et al., 2009). PolyP is also found in archaea and eukaryotes (Lander et al., 2013; Orell et al., 2012; Ruiz et al., 2004). In eukaryotic cells, polyP is both secreted and present in the cytoplasm, vacuoles, nucleus, mitochondria and plasma membranes (Beauvoit et al., 1989; Lichko et al., 2006; Livermore et al., 2016; Offenbacher and Kline, 1984; Reusch, 1992; Suess and Gomer, 2016; Urech et al., 1978; Verhoef et al., 2017).

PolyP is present in *D. discoideum* acidocalcisomes (electron-dense acidic Ca^2+^ storage organelles involved in intracellular pH homeostasis and osmoregulation (Docampo et al., 2005; Marchesini et al., 2002), contractile vacuoles, mitochondria, nuclei and the cytoplasm (Gezelius, 1974; Marchesini et al., 2002; Satre et al., 1986). *D. discoideum* also accumulate extracellular polyP (Suess and Gomer, 2016). At high cell densities, the concomitant high extracellular concentrations of polyP inhibit *D. discoideum* proliferation and induce aggregation, the first stage of development (Suess and Gomer, 2016; Suess et al., 2017). PolyP thus appears to be a signal that cells use to sense their local cell density to anticipate starvation. *D. discoideum* cells use the G protein-coupled receptor GrlD to bind and sense polyP (Suess and Gomer, 2016; Suess et al., 2019). In proliferating *D. discoideum* cells, extracellular polyP accumulation is regulated by polyphosphate kinase 1 (Ppk1) and inositol hexakisphosphate kinase A (I6kA) (Suess and Gomer, 2016). Wild-type *D. discoideum* cells accumulate intracellular polyP during starvation (Livermore et al., 2016), and cells lacking Ppk1 are multinucleate and form fewer spores than wild-type (WT) cells (Livermore et al., 2016), suggesting that polyP affects both growth and development.

In this report, we find that extracellular polyP reduces cell membrane fluidity and recycling, and alters the composition of proteins in crude cytoskeletons. Possibly as a result, polyP inhibits exocytosis of ingested food particles in *D. discoideum* cells, which appears to cause cells to retain food when they are about to outgrow their food supply and starve. This mechanism thus allows polyP to simultaneously promote growth and inhibit proliferation.

## RESULTS

### PolyP inhibits exocytosis in *D. discoideum*

**Fig. 1.**
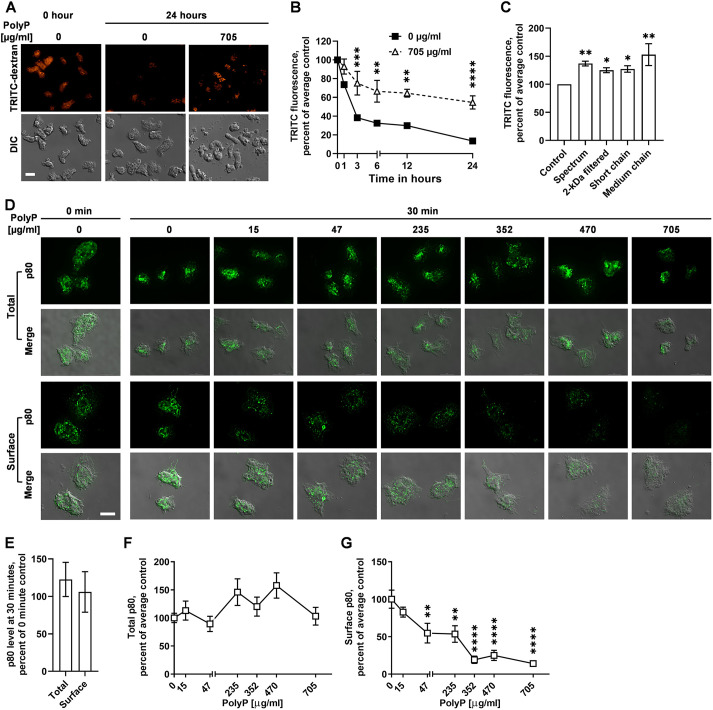
**PolyP inhibits exocytosis in *D. discoideum*.** (A) WT *D. discoideum* cells were incubated with TRITC–dextran in the absence (0) or presence of 705 µg/ml polyP, the uningested TRITC-dextran was removed by washing, and cells were imaged at 0 and 24 h. DIC, differential interference contrast. Scale bar: 10 µm. Images are representative of three independent experiments. (B) Quantification of TRITC–dextran fluorescence from A. The average of no polyP (0) was set at 100%. (C) TRITC–dextran fluorescence per cell in the absence or presence of 705 µg/ml of the indicated polyP at 30 min. (D) Immunofluorescence images of cells stained for p80 (green), with fixed and permeabilized cells (top two rows), and fixed but not permeabilized cells (bottom two rows). Scale bar: 10 µm. Images are representative of three independent experiments. (E) Fluorescence intensity from permeabilized cells (total p80) or non-permeabilized cells (surface p80) at time 30 min in D was normalized to fluorescence intensity from permeabilized cells (total p80) or non-permeabilized cells (surface p80) at time 0 min, respectively. (F) Quantification of fluorescence intensity from permeabilized cells (total p80) in D. (G) Quantification of fluorescence intensity from non-permeabilized cells (cell surface p80) in D. In F and G, the fluorescence intensity of p80 with no polyP (0) was set at 100%. All values in B,C,E,F and G are mean±s.e.m. of three independent experiments. *P<0.05, **P<0.01, ***P<0.001, ****P<0.0001 comparing polyP to no polyP at each time in B, compared to control in C, and compared to 0 polyP in G [Šídák's multiple comparisons test (B), Holm–Šídák's multiple comparisons test (C,D)].

*D. discoideum* cells accumulate extracellular polyP as their cell density increases, and the extracellular polyP concentration (≥470 µg/ml), characteristic of a high cell density, inhibits *D. discoideum* cell proliferation by inhibiting cytokinesis without affecting the growth of the cells (Suess and Gomer, 2016). This then causes cells to be larger and have more protein per cell at stationary phase than in mid-log phase (Soll et al., 1976), and thus to have more stored nutrients in anticipation of starvation. Another possible way for cells to store nutrients is to prevent digestion of endocytosed nutrients and/or prevent excretion (by exocytosis) of partially digested nutrients. *D. discoideum* cells can endocytose dextran, a non degradable fluid phase marker (Hacker et al., 1997; Klein and Satre, 1986; Rivero and Maniak, 2006), and the dextran is then exocytosed, typically after 15–30 min (Klein and Satre, 1986). To determine whether polyP promotes retention of ingested material, cells were exposed to a 30 min pulse of tetramethylrhodamine isothiocynate (TRITC)–dextran and were then washed free of extracellular TRITC–dextran. PolyP concentrations greater than or equal to 470 µg/ml increased retention of ingested TRITC–dextran after 30 min of exocytosis (Fig. 1A,B; Fig. S1A–C). Other sources and preparations of polyP, including 2-kDa filtered polyP, short chain polyP (<60-mer) medium chain (∼100-mer) and 60-mer polyP, also increased the retention of ingested TRITC–dextran (Fig. 1C; Fig. S1D). *D. discoideum* cells require GrlD to bind polyP (Suess et al., 2019), and require Ppk1 and i6kA to accumulate extracellular polyP (Suess and Gomer, 2016). To test whether polyP uses a signal transduction pathway to induce retention of TRITC–dextran, cells lacking GrlD (*grlD^−^*), Ppk1 (*ppk1^−^*), I6kA (*i6kA^−^*) and *i6kA*-null cells overexpressing I6kA (*i6kA^−^/i6kA*) were assayed. Compared to WT cells, 470 and 705 µg/ml polyP did not increase the retention of TRITC–dextran in *grlD^−^*, *ppk1^−^*, and *i6kA^−^* cells, but the effect of polyP was partially rescued in *i6kA^−^/i6kA* cells (Fig. S1E,F), suggesting that polyP uses a signal transduction pathway involving GrlD, Ppk1 and I6kA to inhibit exocytosis. To determine whether the increased retention of TRITC-dextran in WT and *i6kA^−^/i6kA* cells was due to increased macropinocytosis of TRITC–dextran, WT, *grlD^−^*, *ppk1^−^*, *i6kA^−^* and *i6kA^−^/i6kA* cells were incubated with TRITC–dextran, and the levels of ingested TRITC–dextran were measured. PolyP at 470 and 705 µg/ml decreased macropinocytosis in WT but did not significantly alter macropinocytosis in *grlD^−^*, *ppk1^−^* and *i6kA^−^* cells (Fig. S1G,H). Overexpressing I6kA in *i6kA^−^/i6kA* cells rescued the phenotype at 470 but not 705 µg/ml (Fig. S1G,H), suggesting that polyP uses a signal transduction pathway involving GrlD and Ppk1, and possibly I6kA to inhibit macropinocytosis. Together, the data suggest that polyP decreases ingestion, but increases retention of ingested food. At 470 µg/ml polyP, the ingestion decreases by 29% and the retention increases by 32%, whereas at 705 µg/ml polyP the ingestion decreases by 14% and the retention increases by 21%, which would cause the net mass of the cell to increase over time, which has been previously observed (Suess and Gomer, 2016; Yarger et al., 1974).

Extracellular polyP at concentrations ranging from 5 to 15 µg/ml, which correspond to the concentrations of polyP in medium cell density cultures, inhibit the killing of ingested *Escherichia coli* (*E. coli*) in *D. discoideum* cells without significantly affecting the ingestion of *E. coli* or fluorescently labeled heat-killed yeast (zymosan) bioparticles (Rijal et al., 2020). As previously observed for 15 µg/ml polyP, 47, 470 and 705 µg/ml polyP inhibited the killing of ingested *E. coli* by *D. discoideum* cells at 24 h after ingestion (Fig. S1I). 705 µg/ml polyP also reduced the number of ingested zymosan bioparticles (Fig. S1J).

Together, these data suggest that the extracellular polyP concentrations characteristic of high cell densities somewhat inhibit endocytosis and phagocytosis, more strongly inhibit exocytosis, and also inhibit the killing of ingested *E. coli*. The net result is that high extracellular polyP concentrations cause a retention, and to some extent preservation, of ingested nutrients.

During exocytosis, late endosomes fuse with the plasma membrane and form transient exocytic membrane microdomains (Charette and Cosson, 2006). These microdomains are enriched with the transmembrane protein p80, and thus p80 at the plasma membrane can be used to monitor exocytosis in *D. discoideum* (Charette and Cosson, 2006). To determine whether polyP causes the retention of TRITC–dextran by inhibiting exocytosis, we examined total and cell surface p80. The levels of p80 did not change when cells were incubated for 30 min in the absence of polyP (Fig. 1D,E). Exposure of cells to polyP for 30 min did not significantly affect levels of total p80, but 47 µg/ml and higher polyP decreased surface p80 levels (Fig. 1D–F), suggesting that polyP inhibits the surface localization of p80 in *D. discoideum* cells, possibly due to a reduction in exocytosis.

### PolyP increases the retention of internalized membranes

**Fig. 2.**
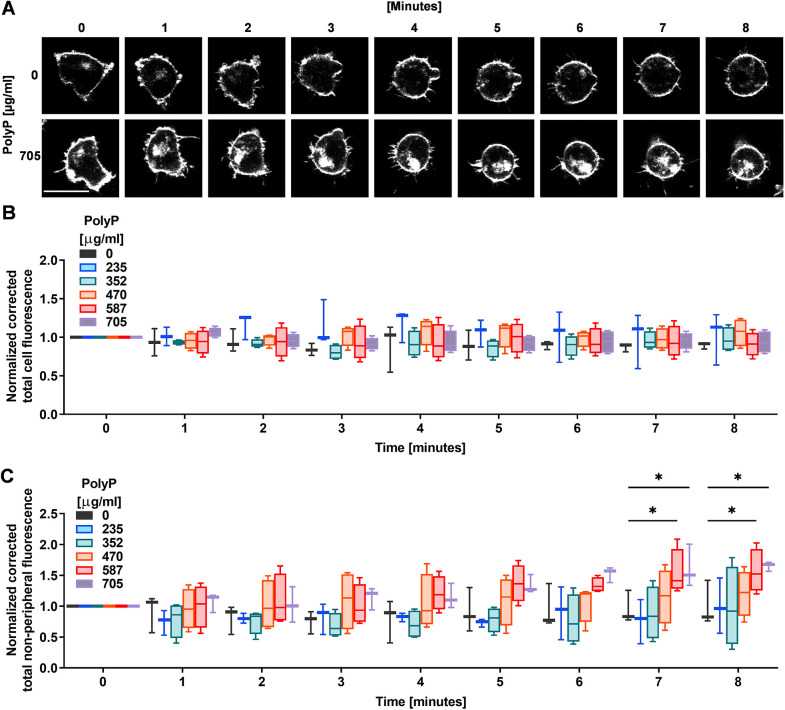
**PolyP increases retention of internalized membranes in the cells.** (A–C) WT *D. discoideum* cells were incubated with the indicated concentration of polyP, stained with membrane dye CellMask Green (gray), and total (A,B) and non-peripheral (A,C) fluorescence intensities were measured at the indicated times. Scale bar: 10 µm. All values are mean±s.e.m. of three independent experiments (B,C). *P<0.05 (Dunnett's multiple comparisons test).

*D. discoideum* cells grow and proliferate in liquid medium and uptake nutrients by pinocytosis (Vines and King, 2019), which requires the continuous internalization of the plasma membrane. However, cells maintain their total surface area by membrane recycling, which involves exocytic fusion of the internalized plasma membrane back to the plasma membrane (Thilo and Vogel, 1980). *D. discoideum* cells replace the entire plasma membrane every ∼45 min by internalization and exocytosis (Thilo and Vogel, 1980). To determine whether polyP-mediated inhibition of exocytosis inhibits cell membrane recycling, we incubated WT cells with polyP for 30 min, stained the plasma membranes were with CellMask Green, a fluorescent lipid analogue, and took images. PolyP did not significantly affect the total cell staining (Fig. 2A,B), whereas a 7–8 min exposure of cell to 587 or 705 µg/ml polyP increased the accumulation of the fluorescent lipid in the interior of cells (Fig. 2A,C). This suggests that polyP increases the retention of internalized cell membranes.

### PolyP might reduce cell membrane fluidity of WT *D. discoideum* cells using GrlD, Ppk1 and I6kA

**Fig. 3.**
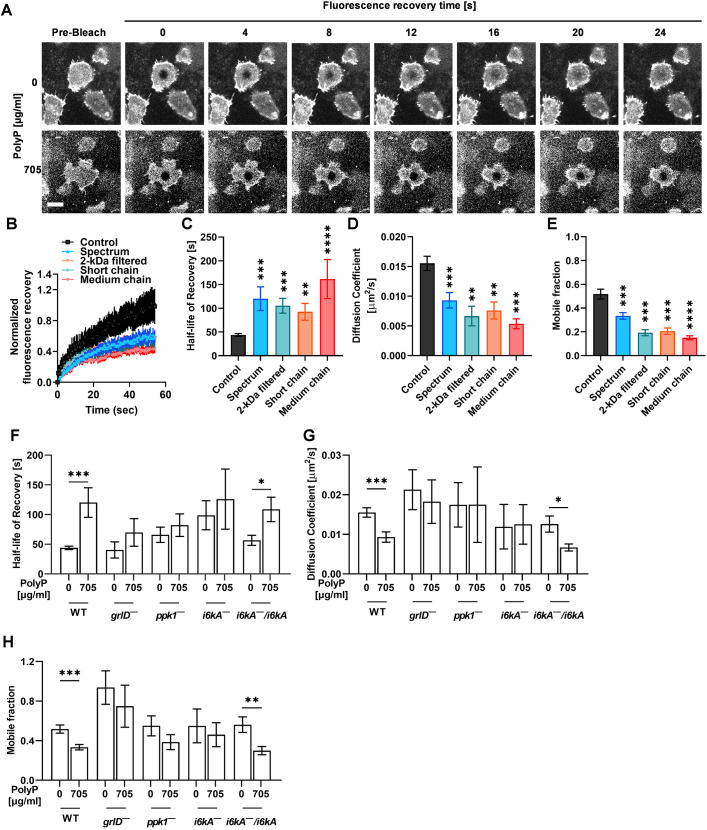
**PolyP reduces the cell membrane fluidity of WT *D. discoideum* cells, and this effect of polyP requires GrlD, Ppk1, and I6kA.** (A) WT *D. discoideum* cells were incubated with the indicated concentration of polyP for 30 min, stained with CellMask Green (gray), photobleached using a 488 nm laser, and fluorescence recovery in the photobleached area was monitored over time. Scale bar: 10 µm. (B) Cells were incubated in the absence (control) or presence of 705 µg/ml of the indicated polyP for 30 min, and fluorescence recovery as in A was measured. Fluorescence intensity at 0 s in the bleached spot after photobleaching was considered 0. (C–E) Half-life of recovery, diffusion coefficient and mobile fraction were generated from the data in B. (F–H) Half-life of recovery, diffusion coefficient, and mobile fraction were calculated for WT, *grlD^−^*, *ppk1^−^*, *i6kA^−^*, and *i6kA^−^/i6kA* cells as in B–E using Spectrum polyP. WT data with and without polyP in C to H are the same, and all the experiments with mutants were done together with WT. All values are mean±s.e.m. of at least three independent experiments. *P<0.05, **P<0.01, ***P<0.001, ****P<0.0001 [Dunn's multiple comparisons test (C–E) and Mann–Whitney test (F–H)].

To investigate whether polyP alters cell membrane fluidity as a mechanism of inhibiting endocytosis and exocytosis, *D. discoideum* cells were stained with the membrane dye CellMask Green, photobleached with a high intensity laser beam, and the recovery of the fluorescence within the photobleached area in cells was measured. Compared to control with no polyP, 705 µg/ml of polyP from Spectrum, 2-kDa filtered, short chain, and medium chain polyP increased the half-life of recovery of fluorescence after photobleaching by ∼2-fold in WT *D. discoideum* cells (Fig. 3A–C; Movies 1 and 2), and decreased the diffusion coefficient (Fig. 3D). The area beneath the curve in Fig. 3B describes the mobile membrane fraction. Compared to the control, all polyP types reduced the membrane mobile fraction (Fig. 3E). Spectrum polyP caused similar effects at concentrations ≥470 µg/ml (Fig. S2A). Together, these data suggest that high cell density polyP (≥470 µg/ml) levels might reduce the cell membrane fluidity of WT *D. discoideum* cells, and that the effect of polyP does not depend on chain length, source or purity of the polyP. However, we cannot exclude the possibility that the delayed fluorescence recovery after polyP treatment could be the consequence of damage or injury to cells caused by photobleaching.

The conditioned medium (CM) from cells at high cell densities contains polyP (Suess and Gomer, 2016). To determine whether CM can mimic the effect of exogenous polyP on membrane fluidity, CM was collected from high cell density (>15×10^6^ cells/ml) WT cell cultures, and dilutions of CM were added to mid-log phase cells. Similar to exogenous polyP, ≥60% CM increased the half-life of recovery, and decreased the diffusion coefficient and mobile fraction in WT cells (Fig. S2B), indicating that a factor present in the CM from cultures at high cell densities reduces cell membrane fluidity.

To determine whether polyP inhibits membrane fluidity using a signal transduction pathway, we tested the effect of polyP on cell membrane fluidity of *grlD^−^*, *ppk1^−^*, *i6kA^−^* and *i6kA^−^/i6kA* cells. Compared to the control with no polyP, 705 µg/ml polyP did not significantly change the half-life of recovery, diffusion coefficient, and mobile fraction of *grlD^−^*, *ppk1^−^* and *i6kA^−^* cells (Fig. 3F–H). However, polyP increased the half-life of recovery, and decreased the diffusion coefficient and mobile membrane fraction of *i6kA^−^/i6kA* cells (Fig. 3F–H). Interestingly, compared to WT cells, *i6kA^−^* cells showed an increase in the half-life of recovery and a decrease in the diffusion coefficient, indicating inherently decreased membrane fluidity (Fig. S2C,D). Similar to what was seen with polyP, 100% CM did not significantly affect the cell membrane fluidity of *grlD^−^*, *ppk1^−^* and *i6kA^−^* cells (Fig. S2C–E), but increased the half-life of recovery and decreased the diffusion coefficient and mobile fraction of *i6kA^−^/i6kA* cells (Fig. S2D,E), suggesting that exogenous polyP or high cell density CM uses a signal transduction pathway involving GrlD, Ppk1 and I6kA to reduce membrane fluidity in *D. discoideum* cells.

### PolyP does not alter random cell motility, speed and cytoskeletal actin, but reduces directionality and increases the formation of filopodia

**Fig. 4.**
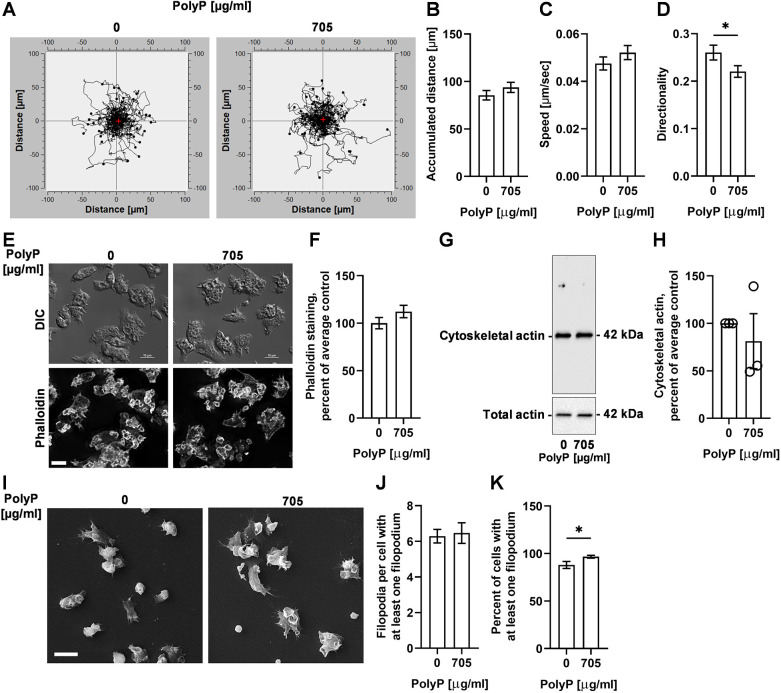
**PolyP does not alter random cell motility, speed and cytoskeletal actin, but reduces directionality, and increases the percentage of the cells with filopodia.** (A) WT cells in the absence (0) or presence of (705 µg/ml) polyP were filmed for 30 min; 30 cells per experiment were tracked, and tracks were graphed. Red plus sign indicates the center of mass after 30 min. The tracks are a compilation of three independent experiments with at least 30 tracks per experiment. (B–D) Quantifications of the effect of polyP from A on cell displacement (accumulated distance), speed, and cell persistence (directionality) over 30 min. (E) Differentital interference contrast (DIC) and fluorescence images of WT *D. discoideum* cells cultured for 30 min in the presence of the indicated concentration of polyP, and then stained with phalloidin (gray) for F-actin. Images are representative of three independent experiments. Scale bar: 10 µm. (F) Quantification of mean fluorescence intensity of phalloidin in E. The average of no polyP (0) was set at 100%. (G) WT cells were incubated with the indicated concentration of polyP for 30 min, and western blots of whole cell lysates or detergent-insoluble cytoskeletons were stained with anti-actin antibodies. Molecular masses in kDa are shown on the right. Blots are representative of three independent experiments. (H) Densitometry was used to estimate levels of polymerized actin. Polymerized actin densitometry was normalized to the total actin. The average of no polyP (0) was set at 100%. (I) Scanning electron micrographs of WT cells cultured the indicated concentration of polyP for 30 min. Images are representative of three independent experiments. Scale bar: 10 µm. (J,K) The effect of polyP on the number of filopodia per cell projecting at least one filopodium (J) and the percentage of cells with at least one filopodium (K). All values are mean±s.e.m. from three independent experiments. *P<0.05 (Mann–Whitney test; D,K).

To determine whether polyP-mediated reduced cell membrane fluidity affects cell motility, WT cells in the absence or presence of 705 µg/ml polyP were tracked for 30 min, and the accumulated distance (total distance travelled along its path), speed (accumulated distance divided by time) and directionality (the straight-line distance between the start and end of the movement of a cell divided by the accumulated distance) of cells were measured. Although polyP did not significantly affect accumulated distance and speed (Fig. 4A–C), polyP reduced the directionality (Fig. 4D). However, cells incubated with 100% CM showed reduced accumulated distance, speed and directionality (Fig. S3A–D), which might be due to the effect of unknown factors in the CM.

To determine whether extracellular polyP affects cytoskeletal actin to reduce cell membrane fluidity, WT cells were incubated with or without 705 µg/ml polyP for 30 min. The cells were then fixed and stained with phalloidin to determine the level of cytoskeletal actin. PolyP did not discernably change the distribution of, or significantly change the levels of, phalloidin staining (Fig. 4E,F). The polyP also did not alter the level of cytoskeletal actin as assayed by western blots of crude cytoskeletons stained for actin (Fig. 4G,H). Unlike 705 µg/ml polyP, 100% CM reduced phalloidin staining and cytoskeletal actin levels (Fig. S3E–H), which, as above, might be due to the effect of unknown factors in the CM. Together, these data suggest that polyP does not alter cytoskeletal actin whereas it does reduce cell membrane fluidity.

Filipodia are actin-rich protrusions involved in sensing the environment and cell anchorage on a surface (Heid et al., 2005; Wood and Martin, 2002). To determine whether extracellular polyP affects filopodia while reducing cell membrane fluidity, we incubated WT cells with 0 or 705 µg/ml polyP for 30 min, and examined filopodia using scanning electron microscopy of fixed cells. PolyP slightly increased the percentage of cells having at least one filopodium without affecting the number of filopodia per cell in the cells that did have filopodia (Fig. 4I–K). Together, these results indicate that, in addition to reducing cell membrane fluidity, polyP slightly decreases directionality of cell movement and causes an increase in the number of cells with actin-rich filopodia, but does not significantly affect cell speed or cytoskeletal actin.

### PolyP alters the proteins associated with the cytoskeleton

In eukaryotic cells, cytoskeletal proteins can link cell membranes, internal vesicles and nuclear membranes. Some properties of the cell membrane, such as membrane fluidity, are also affected by membrane cytoskeletal proteins (Kapus and Janmey, 2013; Lucero and Robbins, 2004). To determine whether polyP alters cytoskeletal proteins, WT cells were incubated with or without 705 µg/ml polyP for 30 min and lysed with 1% Triton X-100. The Triton X-100 insoluble fractions (TIFs) were then assessed through proteomics assays. PolyP significantly increased the accumulation of 80 proteins that fall under the group possessing oxidoreductase activity and 356 proteins under the catalytic activity group, based on gene ontology by molecular function (Table S1). PolyP significantly increased 344 membrane proteins and 666 cellular anatomical entity proteins, and significantly decreased 637 intracellular anatomical structural proteins in the samples (Table S1). Among the detected proteins, polyP significantly increased the abundance of at least three known lipid raft proteins and reduced at least four lipid raft proteins (Table S2) (Barylko et al., 2009; Eckert and Muller, 2009; Funatsu et al., 2000; Otto and Nichols, 2011; Rauchenberger et al., 1997; Wang and Schey, 2015; Wienke et al., 2006). Together, although polyP does not affect levels of cytoskeletal actin (Fig. 4E–H), we found that polyP alters levels of some lipid raft proteins in addition to cytoskeletal proteins that are also associated with the membrane in the TIFs, suggesting that polyP might affect cell physiology, possibly by regulating membrane cytoskeletal proteins.

## DISCUSSION

**Fig. 5.**
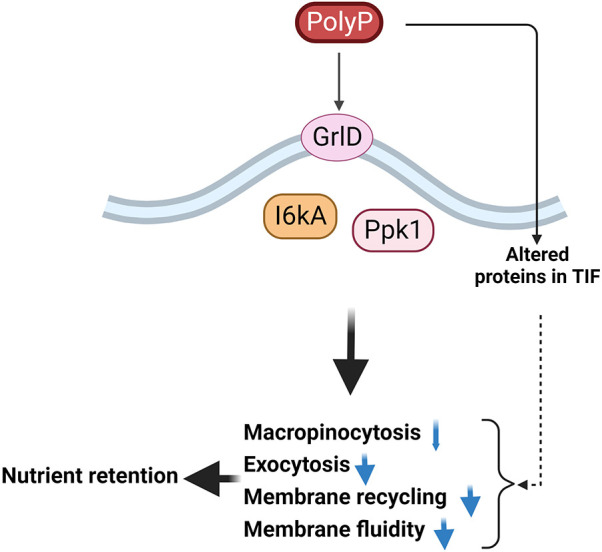
**Summary of polyP signal transduction.** *D. discoideum* cells accumulate polyP as the local cell density increases. PolyP requires GrlD, I6kA and Ppk1 to inhibit macropinocytosis, exocytosis, membrane recycling and possibly membrane fluidity, possibly to retain ingested nutrients in anticipation of starvation. PolyP also alters proteins of the crude cytoskeleton, which might alter membrane fluidity and membrane recycling. The thin blue arrow indicates a slight inhibition; the thick blue arrows indicate greater inhibition. Dashed arrow indicates that alteration of proteins associated with the cytoskeleton in the TIF might reduce endocytosis, exocytosis, membrane recycling or membrane fluidity. The proposed pathway diagram was created using BioRender.com.

Proliferating *D. discoideum* cells accumulate extracellular polyP when their local cell density increases, and a high concentration of extracellular polyP inhibits proliferation when cells are about to outgrow their food supply (Suess and Gomer, 2016; Suess et al., 2019). Here, we found that extracellular polyP strongly inhibits exocytosis of undigested or partially digested food, but only slightly inhibits the ingestion of food in *D. discoideum*. We envision that this response to extracellular polyP would allow proliferating *D. discoideum* cells to store food rather than digest it when cells are about to starve. A different *D. discoideum* secreted factor called autocrine proliferation repressor AprA also inhibits *D. discoideum* proliferation at high cell densities, but does not significantly affect the growth of the cells (Brock and Gomer, 2005). This suggests that the cells find it important to inhibit proliferation but not growth at high cell densities, and use two different factors to accomplish this. Whether AprA inhibits exocytosis is however unknown. Although polyP reduces cell membrane fluidity via a G protein-coupled receptor signaling pathway and alters the composition of proteins in the detergent insoluble crude cytoskeleton (Fig. 5), how polyP changes the membrane physical properties, and whether this directly inhibits exocytosis, is unclear.

Physical properties of cell membranes, such as membrane fluidity, are critical determinants of efficient endocytosis and exocytosis in mammalian cells (Ben-Dov and Korenstein, 2013; Ge et al., 2010). We found that polyP requires GrlD, Ppk1 and I6kA to reduce macropinocytosis and exocytosis. Possibly, because macropinocytic and exocytic activities require fluidic membrane (Degreif et al., 2019), polyP might require GrlD, Ppk1, and I6kA to reduce membrane fluidity, indicating that polyP uses a signal transduction pathway to alter membrane physical properties and membrane trafficking.

*D. discoideum* cells maintain their cell surface area by coordinating the internalization of the cell membrane and exocytosis of the membrane precursor vesicles (Thilo and Vogel, 1980). Although polyP slightly reduced macropinocytosis, where the internalization of the cell membrane with food particles occurs, polyP inhibited exocytosis to a greater extent. Given that exocytosis involves the removal of undigested food particles to the extracellular space and the delivery of precursor membranes to the cell surface, which involves the translocation of exocytic vesicle marker p80 to the cell surface (Charette and Cosson, 2006), inhibiting exocytosis can cause the cells to accumulate internalized membranes and reduce cell surface translocation of p80. *D. discoideum* cells lacking protein kinase C (*pkcA^−^*) accumulate more extracellular polyP and exhibit reduced pinocytosis, but increased exocytosis (Umachandran et al., 2022), suggesting that pinocytosis and exocytosis are not coupled, and polyP might require PkcA to inhibit exocytosis. Although polyP partially inhibits exocytosis of the vesicles containing partially digested or undigested food, we do not know whether polyP affects other secretory pathways involved in protein secretion that are necessary for cell–cell communication (Buratta et al., 2020). We previously observed that polyP inhibits cytokinesis to increase the percentage of large cells (Suess and Gomer, 2016), and it is possible that *D. discoideum* increases cell size while inhibiting exocytosis and membrane recycling using other mechanisms that add material to the plasma membrane.

At low cell densities, and thus low extracellular polyP concentrations (∼15 µg/ml), ∼0.1% of *D. discoideum* cells stop the killing of ingested *E. coli* without affecting the ingestion of the bacteria (Rijal et al., 2020). At high cell densities, when the polyP concentration reaches ∼705 µg/ml, ∼1.7% of *D. discoideum* cells stop the killing of ingested *E. coli*, and many *D. discoideum* cells stop ingesting zymosan bioparticles and stop exocytosing undigested or partially digested zymosan bioparticles to an even greater extent, suggesting a dose-dependent effect of polyP on the storage of food during starvation. Low concentrations of polyP (500 nM) increase mTOR activity (Wang et al., 2003), and activated mTOR might inhibit autophagy, perhaps as a mechanism to inhibit the killing of ingested bacteria.

Interactions of membranes with cytoskeletal components can affect the assembly and disassembly of the cytoskeleton. Although we did not observe any significant change in the level of filamentous actin after polyP treatment of cells, our results indicate that polyP alters some cytoskeletal proteins associated with lipid rafts and the crude cytoskeleton, suggesting that polyP might alter membrane fluidity, possibly by altering the kinetic stability of the membrane (Grimm et al., 2006), and membrane recycling by altering endocytosis and exocytosis (Ben-Dov and Korenstein, 2013; Ge et al., 2010). Therefore, the high levels of extracellular polyP that allow cells to sense that they are at a high cell density and about to outgrow their food supply and starve might alter cytoskeletal proteins that are associated with the membrane and possibly lipid rafts to inhibit exocytosis and thus store ingested nutrients in anticipation of starvation.

## MATERIALS AND METHODS

### *D. discoideum* cell culture

WT AX2 (DBS0237699) (Fey et al., 2013), *grlD^−^* (DBS0350227) (Tang et al., 2018), *ppk1^−^* (DBS0350686) (Livermore et al., 2016), *i6kA^−^* (DBS0236426) (Luo et al., 2003), and *i6kA^−^/i6kA* (Suess and Gomer, 2016) *D. discoideum* strains were obtained from the *Dictyostelium* Stock Center. Cells were grown at 21°C in a type 353003 tissue culture dish (Corning, Durham, NC, USA) in SIH defined minimal medium (Formedium, Norfolk, UK) or on SM/5 agar [2 g glucose, 2 g bactopeptone (Becton Dickinson, Sparks, MD, USA), 0.2 g yeast extract (Hardy Diagnostics, Santa Maria, CA, USA), 0.2 g MgCl_2._7H_2_0, 1.9 g KH_2_PO_4_, 1 g K_2_HPO_4_ and 15 g agar per liter; see http://www.dictybase.org/) on lawns of *E. coli* DB (DBS0350636) in a type 25384-302 Petri dish (VWR, Radnor, PA, USA). 100 µg/ml dihydrostreptomycin (Cat# D5155; Sigma, St Louis, MO, USA) and 100 µg/ml ampicillin (Cat# A-301-25; GoldBio, St Louis, MO, USA) were used to kill *E. coli* in *D. discoideum* cultures obtained from SM/5 agar (Brock and Gomer, 1999). *grlD*^−^*, ppk1^−^* and *i6kA^−^* cells were grown under selection with 5 µg/ml blasticidin (Cat# B-800-25; GoldBio), and *i6kA^−^/i6kA* cells were grown under selection with 5 µg/ml G418 (Cat# N-6386; Sigma). *D. discoideum* cells from a 80–90% confluent culture in a tissue culture dish were collected using a glass pipette, transferred to 15 ml conical tubes (Falcon, VWR), washed twice with SIH by centrifugation at 500 ***g*** for 5 min, the cell density was measured with a hemocytometer, and 100 µl of cells at 10^6^ cells/ml was transferred to a type 353219 96-well, black/clear, tissue culture treated plate (Corning) to obtain 10^5^ cells per well, or 1 ml was transferred to type 353047 24-well tissue culture plate (Corning) to obtain 10^6^ cells per well. For the proliferation assay, *D. discoideum* cells were grown in liquid shaking culture and cell densities were determined as previously described (Suess and Gomer, 2016).

### Bacterial culture

*E. coli* K-12 (BW25113, CGSC#7636; Baba et al., 2006; Datsenko and Wanner, 2000) were grown at 37°C in Luria–Bertani (LB) broth (Becton Dickinson). *E. coli* DB were grown at 21°C on SM/5 agar.

### Recombinant exopolyphosphatase purification and polyP concentration measurement

Recombinant *S. cerevisiae* exopolyphosphatase (PPX) (Gray et al., 2014; Wurst and Kornberg, 1994) was purified as previously described (Brock and Gomer, 2005). PPX was used for treatment of *D. discoideum* cells and culture supernatants as previously described (Rijal et al., 2020). Extracellular polyP secreted by *D. discoideum* strains was assessed by adding 25 µg/ml of DAPI (Biolegend, San Diego, CA), and measuring fluorescence at 415 nm excitation and 550 nm emission as previously described (Aschar-Sobbi et al., 2008). Culture supernatants were clarified by centrifugation at 12,000 ***g*** for 2 min. PolyP concentrations were determined using polyP standards (Spectrum, Cat# S0169; New Brunswick, NJ), and used for all the assays. PolyP (Spectrum) stocks was prepared in PBM buffer (20 mM KH_2_PO_4_, 1 mM MgCl_2_, 0.01 mM CaCl_2_, pH 6.5; Suess et al., 2017), filter sterilized and used for all assays, as was labeled polyP unless specified in the text. To investigate whether any potential small-molecule contaminants present in the polyP affected the assays, the Spectrum polyP was desalted using a Vivacon 500 2 kDa cutoff spin filter (Sartorius, Bohemia, NY) at 7500 ***g*** for 60 min at room temperature, the retentate was resuspended in PBM and collected in an eppendorf tube, and was labeled 2-kDa filtered polyP. Fractionated polyP (medium chain polyP p100 and short chain polyP) were from Kerafast (Boston, MA, USA). PolyP standards of specific chain lengths (60-mer polyP) were kindly provided by Dr Toshikazu Shiba (RegeneTiss Inc., Japan).

### Resolution of polyP by PAGE and Toluidine Blue staining of polyP in gel

PolyP was resolved by polyacrylamide gel electrophoresis (PAGE) using a 5.5×7.5 cm^2^ 10% polyacrylamide [Acryl/Bis 19:1 40% (w/v) solution; VWR Life Science Seradigm, Randor, PA, USA] gel as previously described (Losito et al., 2009; Smith and Morrissey, 2007). The running buffer was 1× TAE (4.84 g Tris, 1.14 ml glacial acetic acid, and 0.37 g EDTA per liter; all reagents were from VWR Life Science Seradigm, Randor, PA), and the 6× sample buffer was 0.01% Orange G (Thermo Fisher Scientific, Fair Lawn, NJ, USA; 30% glycerol; 10 mM Tris-HCl pH 7.4, and 1 mM EDTA]. PAGE was performed at 100 V for 1 h at room temperature until the Orange G had run through two thirds of the gel. Gels were stained with 0.05% toluidine blue (ThermoFisher Scientific), 20% methanol (VWR) and 2% glycerol for 1 h, destained for 3–4 days with several changes of destaining solution (staining solution without Toluidine Blue), and images were taken in white light using a Bio-Rad scanner (Bio-Rad, Hercules, CA, USA).

### Endocytosis and exocytosis assays

Endocytosis and exocytosis assays with TRITC–dextran (average molecular mass 65,000–85,000 Da) (Cat#T1162; Sigma) were performed as previously described (Rivero and Maniak, 2006), with the following modifications. *D. discoideum* cells were seeded in a 96-well black/clear tissue-culture-treated plate. After 30 min, polyP from a 100 mg/ml stock in PBM was added to the cells and mixed by gentle pipetting. 5 µl of 50 mg/ml TRITC–dextran in SIH was added to a well containing 10^5^ cells and mixed by gentle pipetting, the plates were spun down at 300 ***g*** for 2 min in a Heraeus multifuge X3R centrifuge (ThermoFisher Scientific, Germany), and incubated for 30 min to allow endocytosis of TRITC–dextran. Cells were washed two times by gently removing the medium, and gently adding 200 µl SIH, fixed with 4% paraformaldehyde (PFA) in PBS for 10 min, washed twice with 200 µl of phosphate-buffered saline (PBS), and images were taken with a 100× oil-immersion objective on a Nikon Eclipse Ti2 microscope to measure fluorescence of endocytosed TRITC–dextran. For exocytosis, similar to endocytosis, cells were allowed to endocytose TRITC–dextran for 30 min in the absence of polyP, non-ingested TRITC–dextran was removed by washing twice with 200 µl SIH, and cells were incubated in SIH containing different concentrations of polyP to allow exocytosis of ingested TRITC–dextran. After 30 min of incubation, cells were washed with 200 µl of SIH, fixed, and images were taken to determine the fluorescence of ingested TRITC–dextran. Similarly, bulk exocytosis of TRITC–dextran in the presence of polyP of different sizes and purity was measured as previously described (Rivero and Maniak, 2006). The apparent exocytosis of TRITC–dextran slowed down after 3 h, possibly due to reingestion of exocytosed dextran. To overcome this, cells were diluted after 3 h in SIH to one-tenth of the original density, and were allowed to exocytose TRITC–dextran for 24 h, and fluorescence of the retained TRITC-dextran per cell was measured.

### Bacterial survival assay and phagocytosis

*E. coli* K-12 survival assays were performed as previously described (Rijal et al., 2020), except that polyP was added to the assay after WT *D. discoideum* cells were allowed to ingest *E. coli* for 4 h, and the numbers of viable ingested *E. coli* at 24 h and 48 h were determined. Phagocytosis of Alexa Fluor 594-conjugated zymosan bioparticles (Cat#Z23374; ThermoFisher Scientific) was performed as described in Rijal et al. (2020).

### Immunofluorescence

p80 staining was performed as previously described (Charette and Cosson, 2006). For total p80 staining, *D. discoideum* cells were seeded in a 96-well black/clear tissue culture-treated plate, spun down at 500 ***g*** for 2 min, and the SIH medium was changed for SIH containing polyP. After 30 min of incubation, cells were fixed with 4% PFA for 10 min. In a control, at time 0, the medium supernatant was discarded and cells were fixed with 4% PFA. Cells were washed three times with 200 µl of PBS, and permeabilized with 0.1% Triton X-100 (Alfa Aesar, Tewksbury, MA, USA) in PBS for 5 min. Cells were washed two times with PBS, blocked with 1 mg/ml type 0332 bovine serum albumin (VWR) in PBS for 1 h and washed once with PBS. Anti-p80 antibody (1:200; H161; Developmental Studies Hybridoma Bank) in PBS with 0.1% Tween 20 (PBST; Thermo Fisher Scientific) was added to cells and incubated at 4°C overnight. For surface p80 staining, cells were incubated in medium containing polyP for 30 min, medium supernatant was replaced with ice-cold medium containing polyP and incubated for 5 min, 1 µl of anti-p80 antibody (H161) (Ravanel et al., 2001) was added to the cells and incubated for 10 min, cells were washed twice with ice-cold medium to remove excess unbound antibodies, and fixed with 4% PFA for 10 min. Cells stained for total or surface p80 were washed three times with PBST, and incubated with 1:500 Alexa Fluor 488 anti-rabbit-IgG (Jackson Immunoresearch, West Grove, PA, USA) in PBST for 1 h. Cells were washed three times with PBST and 200 µl of PBS was then added to the well. For phalloidin staining, cells incubated with polyP for 30 min were fixed, permeabilized, and stained with 1:2000 ifluor 555-conjugated phalloidin (#ab176756, Abcam, Cambridge, MA, USA) in PBS in the dark for 30 min. Cells were washed three times with PBS before taking images. Each washing step was done for 5 min and all steps were performed at room temperature if not indicated otherwise. Images of cells were taken with a 100× oil-immersion objective on a Nikon Eclipse Ti2 (Nikon), and deconvolution of images was done using the Richardson–Lucy algorithm (Laasmaa et al., 2011) in NIS-Elements AR software. Fluorescence intensity of p80 was analyzed by Fiji (ImageJ; NIH).

### Cell membrane recycling

Cell membrane staining was performed as previously described (Tanaka et al., 2017). To observe the cell membrane, WT *D. discoideum* cells were seeded in a 96-well black/clear tissue culture-treated plate, and spun down at 500 ***g*** for 2 min. Cells in SIH medium were incubated for 30 min in the absence or presence of polyP, and SIH was replaced with SIH containing 1.25× CellMask Green stain (Cat#C37608; Invitrogen). At 10 min after staining, cells were washed twice with SIH medium, incubated with SIH containing the indicated concentration of polyP, and cells were excited by an argon laser (488 nm), and emission was observed through a 515–530 nm band pass filter. Cells were visualized with a 60× water-immersion objective on a FV1000 confocal microscope (Olympus, Center Valley, PA) equipped with Olympus Fluoview Ver.4.2a software, and time-lapse images were taken for 8 min with an interval of 30 s. Fluorescence intensity of the cell or the cell interior was measured using ImageJ software, and corrected total fluorescence was calculated as integrated density−(area of selected cell×mean fluorescence of background readings). The fluorescence intensity at 0 time was set to 1.

### Fluorescence recovery after photobleaching

Photobleaching was performed as previously described (Tanaka et al., 2017). In brief, *D. discoideum* cells were seeded in a 96-well black/clear tissue culture-treated plate, and spun down at 500 ***g*** for 2 min. Cells in SIH medium were incubated for 30 min in the absence or presence of the indicated concentration of polyP, SIH was replaced with SIH containing 1.25× CellMask Green stain (Invitrogen). At 10 min after staining, cells were washed twice with SIH medium, incubated with SIH containing the indicated concentration of polyP, and the full power of the argon laser (488 nm) was applied to a region of a cell for 0.5 s. Cells and the region within the cells to photo-bleach were randomly selected, and were not photo-bleached just at the center of the cells. Images were taken every 0.5 s for 1 min with a 60× water-immersion objective on a FV1000 confocal microscope (Olympus, Center Valley, PA) equiped with Olympus Fluoview Ver.4.2a software. A circular region of interest (ROI) of 3 µm diameter in the cell membrane was bleached, and the fluorescence intensity in the photobleached area (FRAP ROI) was monitored over time. The fluorescence intensity in a 3 µm diameter unbleached region outside of the cell (Base ROI) and a control cell (reference cell ROI) were also monitored. Ten images (10 frames in 5 s) were taken before the FRAP ROI was photobleached to get pre-bleached fluorescence intensities for all ROIs. Normalized fluorescence recovery, half-life of recovery, diffusion coefficient, and mobile fraction were then calculated as described previously (Kappel and Eils, 2004; Tanaka et al., 2017).

### Scanning electron microscopy

For scanning electron microscopy, cells were seeded in 3 ml SIH medium on a glass coverslip on the bottom of a type 353046 six-well plate (Corning) for 30 min, then 705 µg/ml polyP or 100% CM was added to cells and incubated for 30 min, and the medium was gently transferred to an Eppendorf tube containing 120 µl 25% glutaraldehyde (Sigma), mixed, gently added back to the cells, and incubated for 30 min at room temperature. The final concentration of glutaraldehyde was 1%. Cells were rinsed with PBS and dehydrated by successive 30 min incubations with 30%, 50%, 70%, 90%, 100%, 100% and 100% ethanol with a very gentle agitation on a orbital shaker. Cells were not allowed to dry during this procedure. The ethanol was replaced with a ethanol: hexamethyldisilazane (HMDS; Sigma) mixture at a 1:1 and then a 1:3 ratio, and then pure HMDS was added. Each incubation step was 2 h with gentle agitation. All work was done in a fume hood. Pure HMDS was allowed to evaporate overnight to dry the cells. After coating with 20 nm gold using a type 108 sputter coater (Cressington, Redding, CA), the cells were observed with a scanning electron microscope (Tescan Vega, Warrendale, PA).

### Cytoskeletal protein analysis by mass spectrometry

To isolate proteins associated with the cytoskeleton, we followed the method of Lingwood and Simons (2007) with modifications. Briefly, *D. discoideum* cells at 10^6^ cells/ml density were incubated in 10 ml SIH in the absence or presence of 705 µg/ml polyP. After 30 min, cells were collected by centrifugation at 500 ***g*** for 5 min and resuspended in 1 ml TNE buffer (150 mM NaCl, 2 mM EDTA, 50 mM Tris-HCl pH 7.4) containing protease and phosphatase inhibitors (Cell Signaling Technology, Danvers, MA), passed through a 25 gauge needle to shear the cells, and then treated with 1% Triton X-100, and after 30 min of incubation on ice, lysates were centrifuged at 15,000 ***g*** for 1 h at 4°C. For protein content determination, the 1% Triton X-100 insoluble fractions (TIFs) were resuspended in 1× SDS sample buffer containing protease and phosphatase inhibitors, and heated at 95°C for 5 min. Samples were loaded onto a 4–20% polyacrylamide gel, electrophoresed until the Bromophenol Blue in the sample buffer had migrated 5 mm from the bottom of the wells, and then the portion of the gel from the bottom of the well to the dye front was excised with a clean razor blade, diced into 2 mm^2^ cubes, and transferred to Eppendorf tubes pre-rinsed with ethanol. Sample preparation for LC-MS was performed using in-gel protein digestion protocol at the Department of Chemistry mass spectrometry core facility at Texas A&M University (https://mass-spec.chem.tamu.edu/proteomics/proteomics-protocols.php). Mass spectrometry proteomics was performed on a Thermo Scientific Orbitrap Fusion tribrid mass spectrometer equipped with a Dionex UltiMate 3000 reverse-phase nano-UHPLC system. Biological processes, molecular functions and cellular locations were determined for identified proteins using the Gene Ontology resource (http://geneontology.org/), and PANTHER analysis and enrichment analysis was performed using Fisher's exact test and Bonferroni correction for multiple testing. Membrane raft proteins were identified in Triton X-100 insoluble membrane fraction by searching the UniProt database and PubMed.

### Cell motility, speed and directionality measurement

Cells were seeded in 96-well black/clear tissue culture-treated plates, spun down at 500 ***g*** for 2 min, and SIH medium was changed to SIH, SIH containing polyP or 100% CM. At least 30 cells per experiment were imaged every 15 s for 30 min using a 40× objective on a Nikon Eclipse Ti2 (Nikon). Accumulated distance, speed and directionality was calculated as previously described (Phillips and Gomer, 2012).

### Cytoskeletal actin extraction and immunoblotting

Cells were cultured as above, and after 30 min, for whole-cell lysates, the culture medium was removed, and cells were lysed in 75 µl of 1× SDS sample buffer. Actin filaments were extracted as previously described (Rijal et al., 2019). The whole-cell lysate and crude cytoskeletons were resolved on 4–20% Mini-PROTEAN Tris-glycine polyacrylamide gels (Bio-Rad), and western blots were stained with 1:5000 diluted anti-β-actin mouse monoclonal antibody (Cat# 3700S; Cell Signaling Technology) to detect total and cytoskeletal actin as described in Rijal et al. (2019).

### Statistical analysis

Statistical analyses were performed using GraphPad Prism 9 (GraphPad, San Diego, CA). A *P*<0.05 was considered significant.

## Supplementary Material

10.1242/joces.260107_sup1 Supplementary information
