# Supplementary material for: Dictyostelium discoideum cells retain nutrients when the cells are about to outgrow their food source
Source: J Cell Sci. 2022 Sep 21;135(18):jcs260107. doi: 10.1242/jcs.260107 (PMC9592050; doi:10.1242/jcs.260107)
Supplement: Supplementary information [file joces-135-260107-s1.pdf]

Supplementary Figure 1

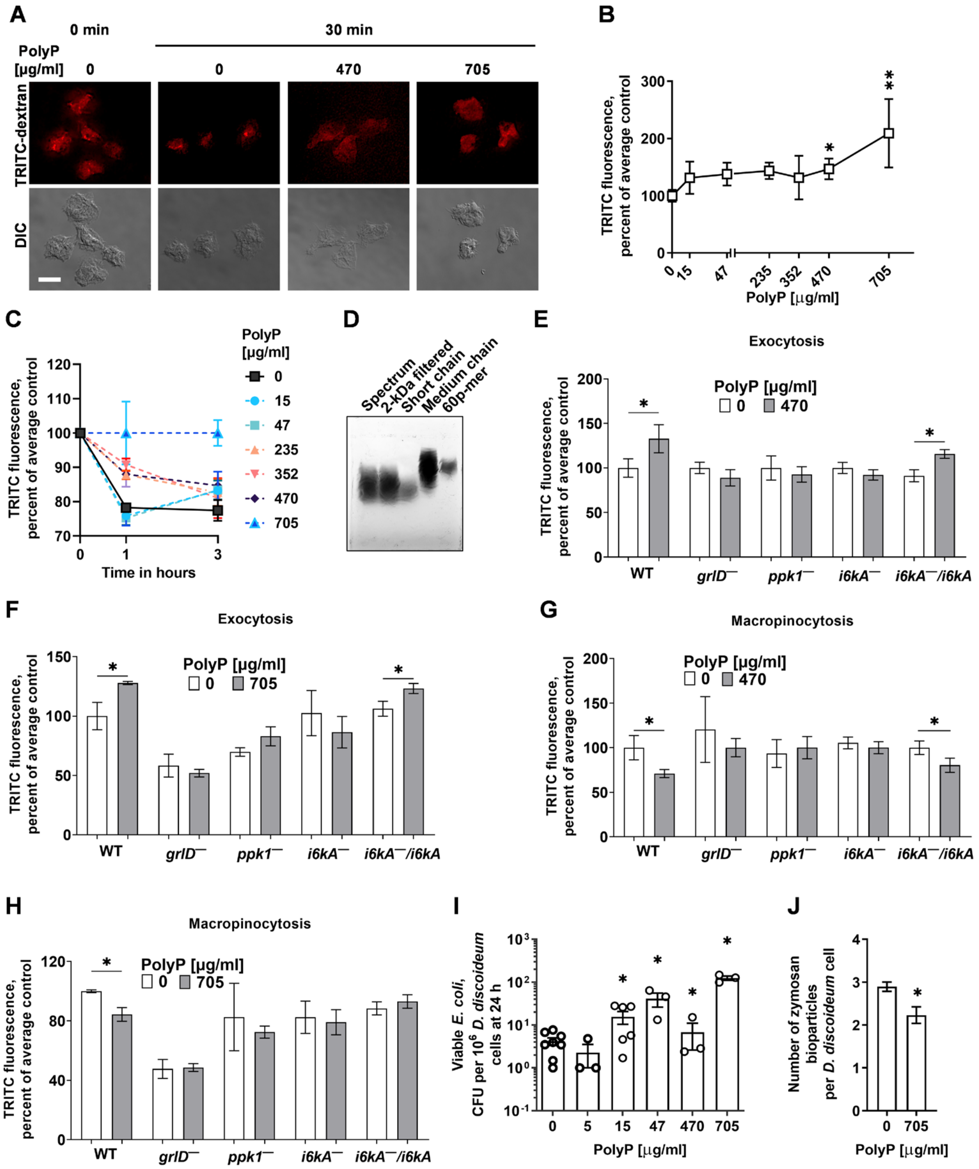

Fig. S1. PolyP inhibits exocytosis, macropinocytosis, and phagocytosis in *D. discoideum* cells. A) WT *D. discoideum* cells were incubated with TRITC-dextran in the absence or presence of polyP, uningested TRITC-dextran was removed by washing, cells were imaged at 0 and 30 minutes. DIC indicates differential interference contrast. Bar is 10 µm. Images are representative of 3 independent experiments. **B)** Quantification of TRITC-dextran fluorescence from **A** after 30 minutes in the presence of the indicated concentrations of polyP. The average of 0 polyP was set

Journal of Cell Science • Supplementary information

to 100%. **C)** TRITC-dextran fluorescence per cell in the absence or presence of increasing concentrations of polyP at 3 hours. The 0 hour value for each polyP concentration was set to 100%. **D)** The indicated polyP samples were electrophoresed on a 10% polyacrylamide gel, which was then stained with toluidine blue. 60-mer polyP (60p-mer) standard was used as a standard. **E-H)** Exocytosis (E and F) or macropinocytosis (G and H) of TRITC-dextran. The average of wild-type (control) was considered 100%. **I)** Colony forming unit (CFU) of viable ingested *E. coli* in wild- type *D. discoideum* cells in the presence of the indicated concentrations of polyP at 24 hours. **J)** The number of pHrodo zymosan bioparticles ingested per *D. discoideum* cell in 30 minutes in the presence or absence of 705 µg/ ml polyP was determined. All values are mean ± SEM of at least 3 (B, C, E-J) independent experiments. * indicates $p < 0.05$, ** $p < 0.01$ (One-way ANOVA with Fisher's LSD test (B), Mann Whitney test (E-J)).

Journal of Cell Science • Supplementary information

Supplementary Figure 2

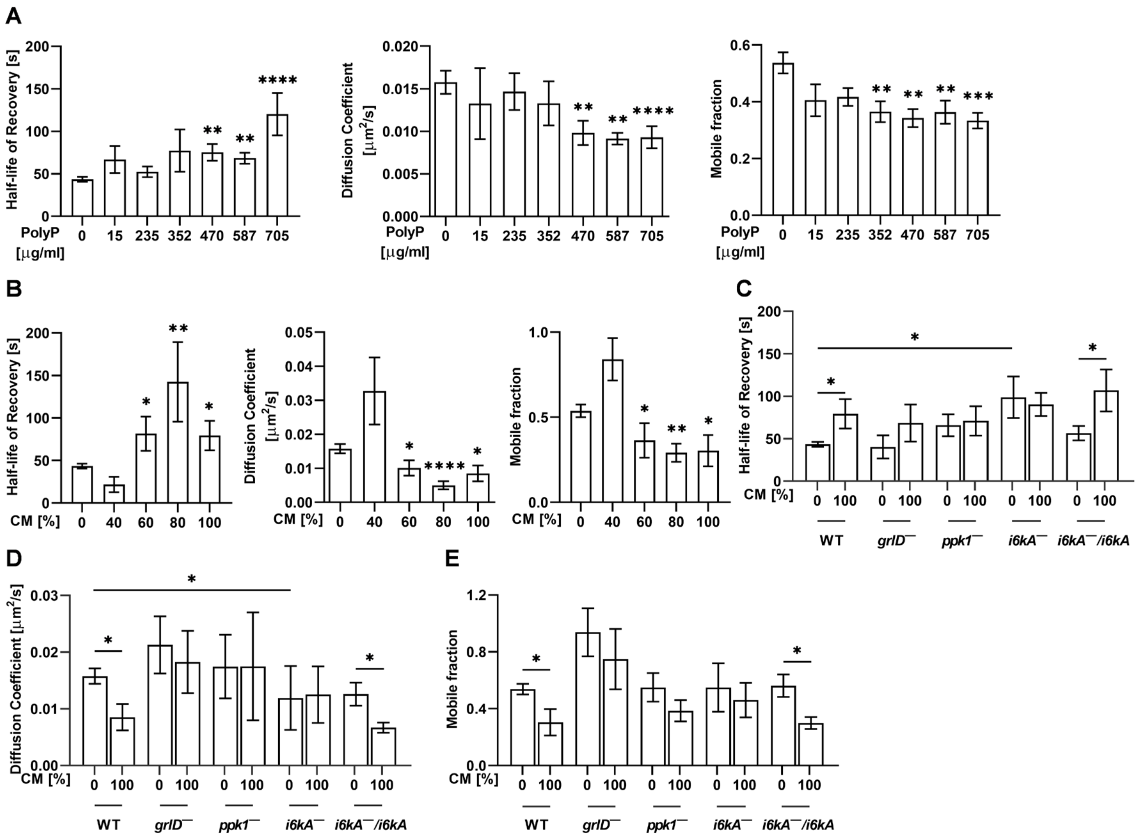

Fig. S2. PolyP or high cell density WT conditioned medium (CM) reduces the cell membrane fluidity of wild-type *D. discoideum* cells, and this effect of polyP or CM requires GrlD, Ppk1, and I6kA. A) WT *D. discoideum* cells were incubated with the indicated concentrations of polyP for 30 minutes, and the half-life of recovery, diffusion coefficient and mobile fraction were calculated as in Figure 3. **B)** The half-life of recovery, diffusion coefficient and mobile fraction were determined as in (A), but in the presence of conditioned medium (CM). **C-E)** The half-life of recovery, diffusion coefficient and mobile fraction of the indicated *D. discoideum* strains with no CM (0) or in the presence of 100% CM were determined. All values are mean ± SEM of at least 3 independent experiments. * $p < 0.05$, ** $p < 0.01$, *** $p < 0.001$, **** $p < 0.0001$ (One-way ANOVA with Fisher's LSD test compared to 0 polyP (A and B) and Mann Whitney test (C-E)).

Journal of Cell Science • Supplementary information

Supplementary Figure 3

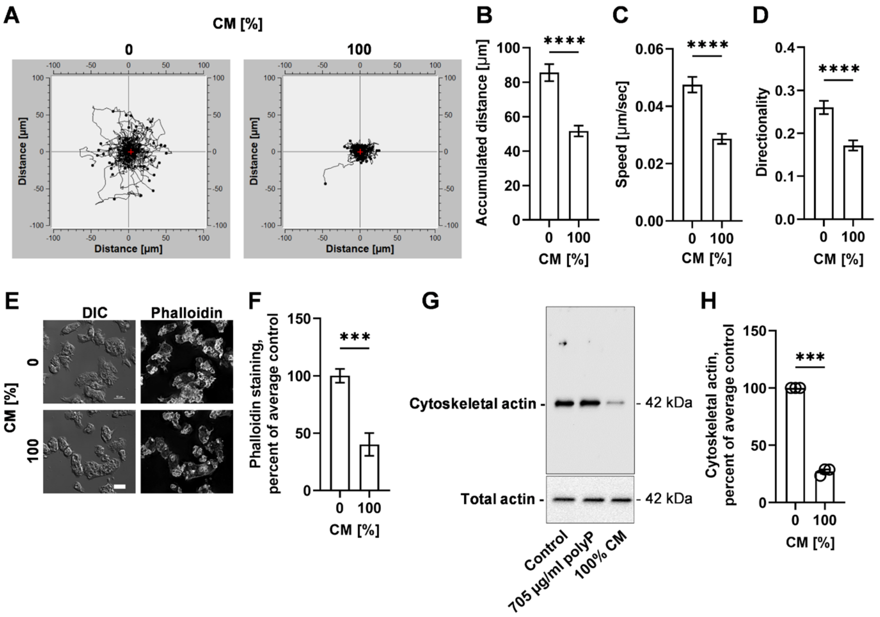

Fig. S3. Conditioned medium effects on cells. A) WT cells in the absence (0) or presence (100%) of conditioned medium (CM) were filmed for 30 minutes; at least 30 cells per experiment were tracked, and tracks were graphed. Red plus sign indicates the center of mass after 30 minutes. The tracks are a compilation of three independent experiments with at least 30 tracks per experiment. **B-D)** Quantifications of the effect of CM from A on cell displacement (accumulated distance), speed, and cell persistence (directionality) over 30 minutes. **E)** Differentital interference contrast (DIC) and fluorescence images of WT *D. discoideum* cells stained with phalloidin (gray) for F-actin in the absence (0) or in the presence (100%) of CM are shown. Images are representative of 3 independent experiments. Bar is 10 µm. **F)** Quantification of mean fluorescence intensity of phalloidin in **E** is shown. The average of no CM (0) was considered 100%. **G)** WT *D. discoideum* cells were incubated with 0% or 100% CM, and western blots of whole cell lysates or detergent-insoluble cytoskeletons were stained with anti-actin antibodies. Molecular masses in kDa are at right. Blots are representative of three independent experiments; blot is the same as used for Figure 4G. H) Densitometry was used to estimate levels of polymerized actin. Polymerized actin densitometry was normalized to the total actin. Average of no CM (0) was considered 100%. All values are mean ± SEM from 3 independent experiments. For B, C, D, F, and H, *** $p < 0.001$, **** $p < 0.0001$ (Mann Whitney test).

Journal of Cell Science • Supplementary information

Table S1. Enrichments in proteins in Triton X-100 insoluble membranes from WT *D. discoideum* cells treated with 705 µg/ml polyP. WT *D. discoideum* cells were cultured with or without 705 µg/ml polyP for 30 minutes. The Triton X-100 insoluble material was then analyzed by proteomics. Gene Ontology analysis was performed on proteins overabundant or underabundant. Bonferroni corrected fold enrichment for molecular functions and cellular components with $p < 0.05$ are listed.

Supplementary Table 1

Overabundant proteins in polyP treated wild-type cells

	Number of proteins	Fold Enrichment	P value
GO molecular function complete			
Oxidoreductase activity	80	1.7	0.0196
Catalytic activity	356	1.22	0.0304
GO cellular component complete			
Membrane	344	1.23	0.0188
Cellular anatomical entity	666	1.14	0.00212

Underabundant proteins in polyP treated wild-type cells

	Number of proteins	Fold Enrichment	P value
GO cellular component complete			
Intracellular anatomical structure	637	1.14	0.046

Journal of Cell Science • Supplementary information

Table S2. Polyphosphate-induced changes in known lipid raft proteins in TIF. WT *D. discoideum* cells were cultured with or without 705 µg/ml polyP for 30 minutes. The TIF was then analyzed by proteomics. Known lipid raft proteins that are overabundant or underabundant after polyP treatment are listed.

Supplementary Table 2

Overabundant lipid raft proteins			Underabundant lipid raft proteins		
**Accession ID**	**Proteins**	**P-value**	**Accession ID**	**Proteins**	**P-value**
P54677	Phosphatidylinositol 4-kinase	<0.05	Q54WZ2	Vacuolin-B	<0.05
Q54DL7	von Willebrand factor A domain-containing protein	<0.05	O96923	Gelsolin-related protein of 125 kDa	<0.05
P36412	Ras-related protein Rab-11A	<0.05	Q9GPM4	Phosphoglycerate kinase	<0.05
			Q54ET2	Presenilin-A	<0.05

Journal of Cell Science • Supplementary information

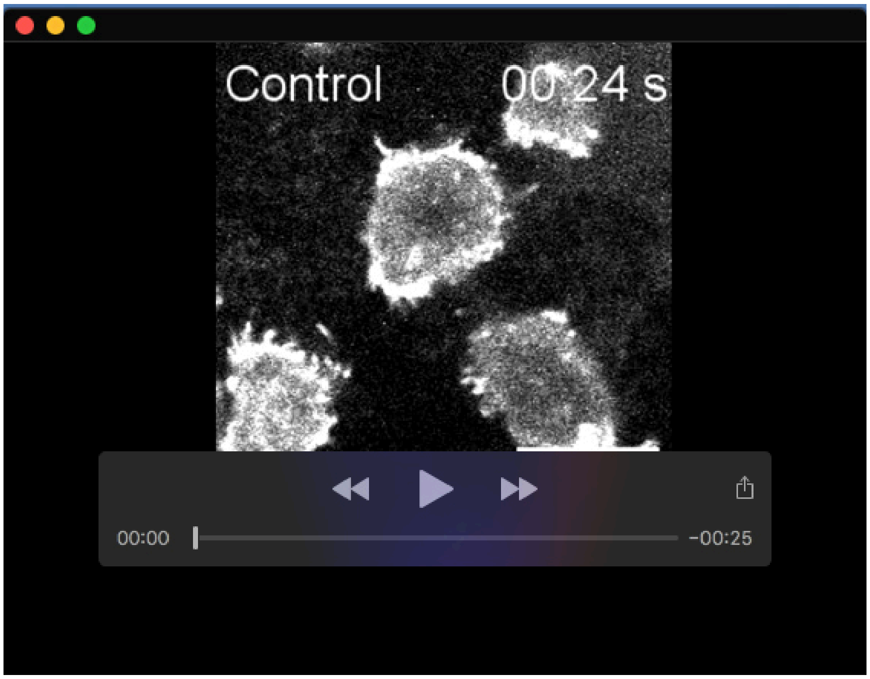

Movie 1. Live-cell microscopy showing fluorescence recovery after photobleaching of WT *D. discoideum* cells. WT *D. discoideum* cells were incubated in SIH without polyP, and fluorescence recovery after photobleaching was monitored over time. Fluorescence images were collected every 0.5 seconds for 59 seconds using a 60× water-immersion objective. Bar is 10 µm. Indicated time after bleaching is in minutes:seconds.

Journal of Cell Science • Supplementary information

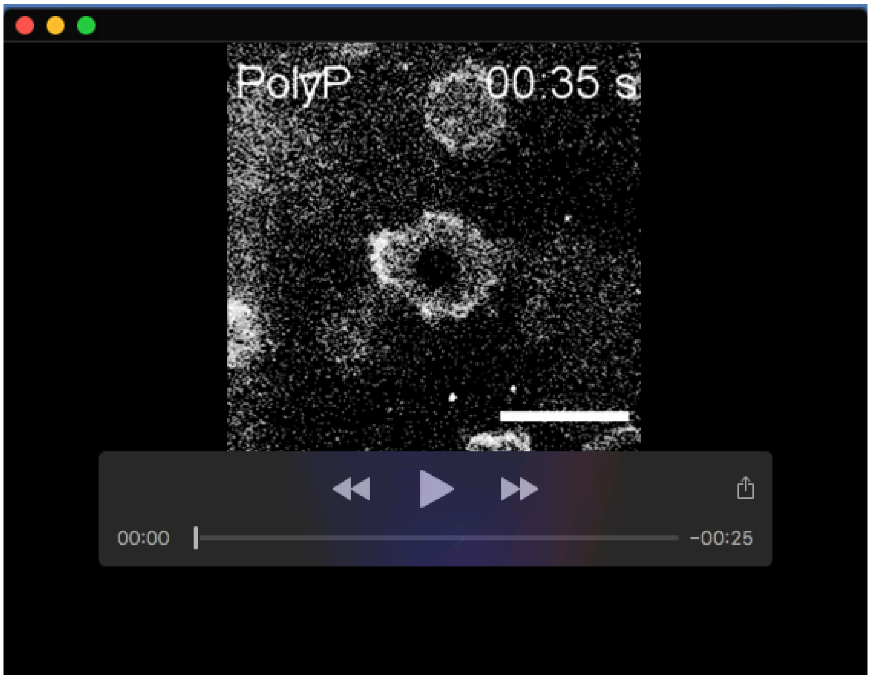

Movie 2. Live-cell microscopy showing fluorescence recovery after photobleaching of polyP treated WT *D. discoideum* cells. WT *D. discoideum* cells were incubated in SIH with 705 µg/ml polyP, and fluorescence recovery after photobleaching was monitored over time. Fluorescence images were collected every 0.5 seconds for 59 seconds using a 60× water-immersion objective. Bar is 10 µm. Indicated time after bleaching is in minutes:seconds.

Journal of Cell Science • Supplementary information
