# Supplementary material for: Dictyostelium discoideum cells retain nutrients when the cells are about to outgrow their food source
Source: J Cell Sci. 2022 Sep 21;135(18):jcs260107. doi: 10.1242/jcs.260107 (PMC9592050; doi:10.1242/jcs.260107)
Supplement: Supplementary file 1 [file joces-135-260107_review_history.pdf]

Journal of Cell Science

Dictyostelium discoideum cells retain nutrients when the cells are about to outgrow their food source

Ramesh Rijal, Sara A. Kirolos, Ryan J. Rahman and Richard H. Gomer
DOI: 10.1242/jcs.260107

Editor: David Stephens

Review timeline
Original submission: 8 April 2022
Editorial decision: 17 May 2022
First revision received: 19 June 2022
Editorial decision: 25 July 2022
Second revision received: 17 August 2022
Accepted: 18 August 2022

Original submission

First decision letter

MS ID#: JOCES/2022/260107

MS TITLE: Dictyostelium discoideum cells sense their local density and retain nutrients when the cells are about to overgrow their food source

AUTHORS: Ramesh Rijal, Sara Ann Kirolos, Ryan J Rahman, and Richard Gomer
ARTICLE TYPE: Research Article

We have now reached a decision on the above manuscript.

To see the reviewers' reports and a copy of this decision letter, please go to: https://submit-jcs.biologists.org and click on the 'Manuscripts with Decisions' queue in the Author Area. (Corresponding author only has access to reviews.)

As you will see, the reviewers raise a number of substantial criticisms that prevent me from accepting the paper at this stage. They suggest, however, that a revised version might prove acceptable, if you can address their concerns. If you think that you can deal satisfactorily with the criticisms on revision, I would be pleased to see a revised manuscript. We would then return it to the reviewers.

While there are some significant technical concerns raised by both reviewers, I and reviewer 2 wish to provide some further context on comments from reviewer 1. We found the tone quite terse and some comments bordering on inappropriate. I (as Editor) have decided to convey the reviewer comments to you as they were submitted but provide additional context to hopefully help with your revision. This is included as an attachment to this email. I very much hope this will help you revise the work for resubmission to us.

Please ensure that you clearly highlight all changes made in the revised manuscript. Please avoid using 'Tracked changes' in Word files as these are lost in PDF conversion.

I should be grateful if you would also provide a point-by-point response detailing how you have dealt with the points raised by the reviewers in the 'Response to Reviewers' box. Please attend to

© 2022. Published by The Company of Biologists under the terms of the Creative Commons Attribution License (https://creativecommons.org/licenses/by/4.0/).

all of the reviewers' comments. If you do not agree with any of their criticisms or suggestions please explain clearly why this is so.

Reviewer 1

Advance summary and potential significance to field

The present study by Rijal et al addresses the timely topic of "quorum sensing" in free living eukaryotes. The amoeba D. discoideum represents one of the few truly tractable system to study these phenomena, and the Gomer lab, who has been at the front of this research, has made seminal and exciting discoveries related to the use of extracellular polyP secretion and accumulation as a major driver of proliferation control. The present study extends naturally on previous publications from the group and discovers a potentially new twist on the story namely that (one of the ways) polyP impacts the amoeba by regulating their "appetite and digestion" and modulating the digestion, leading to food retention. This is novel and exciting for the D. discoideum community and beyond.

Comments for the author

Overall, the study makes use of a large and appropriate spectrum of approaches and techniques to decipher the mechanisms of polyP impact on proliferation and phagocytosis, with a strong emphasis on the phagosome maturation characteristics that are affected. Despite this relatively positive evaluation, the manuscript nevertheless feels like a disconnected collection of observations, with sometime relatively "artificial" links and arguments introduced to smooth the transition. A greater effort should be made to integrate the various findings and propose a working model (including in the form of a schematic drawing). The causality link between the observations and the outcome on food retention are not always clear. Especially, the relationship between membrane fluidity, motility, microdomains, raft proteins ... is not straightforward, even more so in the light of the criticisms concerning lipidomic and proteomic investigations (see below).

Major comments:
1- I do not understand how it is possible to obtain MS lipidomcic information from a detergent extract. This is reputed difficult to impossible. Either there are caveats, or the Materials and Methods is vastly incomplete.
2- The lipidomic analyses include lipid species that are NOT membrane components, such as TAGs, constituents of lipid droplets, and DAGs. This means that the TIF contained lipid droplet material and likely other contaminants. This fully precludes the interpretation proposed about microdomains and rafts etc... In addition, the presence of some of the lipids appears weird for D. discoideum. For example a derivative of cholestanol ... but D. discoideum does not synthesise cholesterol, from which it is probably derived!!! This sis also true for extremely bizarre polymeric and/or cyclic siloxane molecules such as Cyclohexasiloxane, or Cyclopentasiloxane etc... All this, without better explanations and/or metabolic (synthesis/degradation) description sounds like contaminations.
3- The authors analysed the Triton Insoluble Fraction (TIF), as representing the membrane microdomains. But this is only partly correct. The operational definition of "rafts" is Triton Insoluble FLOATING fraction (TIFF), meaning the membrane domains that float on a sucrose gradient. This is absolutely required to separate the cytoskeleton, which fully precipitates together with the TIF. The presence of a gelsolin-related proteins speaks for this problem.
4- In fact, the relatively profound differences reported for lipids appear to me to be exaggerated for a short 30 minutes only treatment with polyP. Most of the lipids reported are not signalling molecules, but metabolic and/or structural lipids.

Minor comments:
1 - The lipid species in Fig 5 "17-Pentatriacontene" is presented twice and with different results.
2- The classification of proteins in Table 3 as lipid raft components is almost fanciful. In addition, lipids are not "up/downregulated", but maybe under/overabundant.
3- In Figures 1F, G and S1B, please, use a linear X axis scale, otherwise the representation is distorted and misleading.
4- Line 314, the argument about uncoupling of endocytosis and exocytosis appear weird. Cells would either shrink or inflate to death!?

© 2022. Published by The Company of Biologists under the terms of the Creative Commons Attribution License (https://creativecommons.org/licenses/by/4.0/).

5- Line 327, autophagy is not (directly) linked to phagocytosis and therefore, not to killing of ingested bacteria.
6- Figure 1D, it is hard to imagine that the technique to detect surface p80 works reliably and quantitatively, because it is known that PFA fixation partially damages and permeabilises the plasma membrane
7- In Fig 3, the FRAP is performed right in the center of the cell, likely affecting the whole cell volume, and not only the plasma membrane.

Reviewer 2

Advance summary and potential significance to field

This paper makes a bold statement, that polyphosphate changes membrane fluidity and thus changes the nutritional state of cells acting through a signalling pathway. It this is true it's a very interesting story.

Comments for the author

The key point to differentiate is between polyphosphate just physically obstructing membrane traffic and thus the author's measure of fluidity, and between the receptor-mediated pathway they're describing. I'd be suspicious that the concentrations of polyphosphate they'd describe would obstruct vesicle traffic.
The authors have tested this, explicitly, in Fig 3F-H. These figures are extremely important to justify the conclusions. But the statistics shown are wrong to support the point, and the results look worryingly ambiguous. If I understand right, 3C shows that half-time recovery is significantly different between control and 3 different polyphosphate preps. 3F does not show a significant difference when the same test is done on grlD- cells. But (a) the difference is very similar - it's just the error bars look worse in the control for grlD; (b) this is in any case not publishable stats. They need to test whether they can be confident that the ratio is different between grlD and wild type. Getting a significant experiment in one line and not one in the other doesn't mean anything; maybe the experiment on the mutant was less successful for example. They need an explicit test (perhaps using ANOVA?) of difference. (c) the same is true for both of the other measures - it looks like polyP affects grlD- cells a lot in 3G and 3H. (d) the figure legend doesn't say whether the experiments in 3F-H were done with 'spectrum', 'filtered', 'short chain' or 'medium chain' preps; this completely changes the interpretation.
The authors need to do a single experiment (in triplicate or more) comparing WT and grlD (etc) on the same day and the same conditions, then use a sound test to determine whether the effects of the treatment are different.
Another thing I'd question - the authors come to the conclusion that wild type cells are spitting out digestible food, based pretty exlusively on the data in Fig S1I. Those are interesting, but not nearly clear enough to justify the strong conclusions in the text and abstract. Maybe cells eat all their food (you'd think so, biologically) and cell killing reports something different based on membrane fluidity/recycling. I would try and strengthen this data; and in any case I would greatly tone down the conclusions about cells not digesting bacteria they've phagocytosed, perhaps to the discussion, not theresults and abstract.
- are they really measuring fluidity, not micropinocytosis/exocytosis? Please discuss more...
- do you really want to conclude "PolyP alters the protein composition of lipid microdomains" from the data in Fig. 5? That looks like a max 4% difference. It may be stasticially significant, but can that small a difference have any biological relevance?

© 2022. Published by The Company of Biologists under the terms of the Creative Commons Attribution License (https://creativecommons.org/licenses/by/4.0/).

First revision

Author response to reviewers' comments

Reviewer 1 Advance Summary and Potential Significance to Field:
The present study by Rijal et al addresses the timely topic of "quorum sensing" in free living eukaryotes. The amoeba D.discoideum represents one of the few truly tractable system to study these phenomena, and the Gomer lab, who has been at the front of this research, has made seminal and excitingdiscoveries related to the use of extracellular polyP secretion and accumulation as a major driver of proliferation control. The present study extends naturally on previous publications from thegroup and discovers a potentially new twist on the story, namely that (one of the ways) polyP impacts the amoeba by regulating their "appetite and digestion" and modulating thedigestion, leading to food retention. This is novel and exciting.
Overall, the study makes use of a large and appropriate spectrum of approaches and techniques to decipher the mechanismsof polyP impact on proliferation and phagocytosis, with a strong emphasis on the phagosome maturation characteristics that are affected. Despite this relatively positive evaluation, themanuscript nevertheless feels like a disconnected collection of observations, with sometime relatively "artificial" links and arguments introduced to smooth the transition. Agreater effort should be made to integrate the various findings and propose a working model (including in the form of a schematic drawing). The causality link between theobservations and the outcome on food retention are not always clear. Especially, the relationship between membrane fluidity, motility, microdomains, raft proteins ... is not straightforward, evenmore so in the light of the criticisms concerning lipidomic and proteomic investigations (see below).
Reply: Thank you for your nice comments. We have included a summary as Figure 6 to integrate our various findings.

Major comments:

1- I do not understand how it is possible to obtain MS lipidomcic information from a detergent extract. This is reputed difficultto impossible. Either there are caveats, or the Materials and Methods is vastly incomplete.
Reply Surprisingly, there is a large body of literature on detergent-insoluble lipids that appear to form detergent-insoluble complexes with the cytoskeleton. These are sometimes called detergent-insoluble lipid rafts (although these rafts do appear to be complexed with cytoskeletal proteins). We used a standard protocol for the preparation of the detergent-insoluble complexes of lipids and cytoskeletal proteins. To clarify the procedure and results, we changed the title of the method in line 892 from "*Lipid microdomain, fatty acid analysis, and lipid and protein analysis*" to "*Lipid extraction, fatty acid analysis, and lipid and protein analysis*", updated and clarified the method for lipid extraction starting on line 533, replaced 'lipid microdomains' with 'crude cytoskeleton' in the abstract (line 32), replaced 'alters the composition of lipid microdomains' in the introduction (line 85) with 'alters the composition of cytoskeleton associated lipids and proteins', replaced 'Triton X-100 insoluble lipid microdomains' with 'Triton insoluble fraction (TIF)' and 'lipid microdomains' with 'cytoskeleton' throughout the text and figures.

2- The lipidomic analyses include lipid species that are NOT membrane components, such as TAGs, constituents of lipid droplets,and DAGs. This means that the TIF contained lipid droplet material and likely other contaminants. This fully precludes the interpretation proposed about microdomains and raftsetc... In addition, the presence of some of the lipids appears weird for D. discoideum. For example a derivative of cholestanol ... but D. discoideum does not synthesise cholesterol,from which it is probably derived!!! This sis also true for extremely bizarre polymeric and/or cyclic siloxane molecules such as Cyclohexasiloxane, or Cyclopentasiloxane etc...
Reply We have now added a reference (Lines 251 to 252) where other workers observe cyclohexasiloxane and cyclopentasiloxane coming from the silica backbone column used for gas chromatography, and that these are indeed contaminants. We have no explanation for the derivative of cholestanol, but it is there in all six samples. We now added a reference on line 254 where people have found steroids in *Dictyostelium*.

© 2022. Published by The Company of Biologists under the terms of the Creative Commons Attribution License (https://creativecommons.org/licenses/by/4.0/).

3- The authors analysed the Triton Insoluble Fraction (TIF), as representing the membrane microdomains. But this is only partlycorrect. The operational definition of "rafts" is Triton Insoluble FLOATING fraction (TIFF), meaning the membrane domains that float on a sucrose gradient. This is absolutely requiredto separate the cytoskeleton, which fully precipitates together with the TIF. The presence of a gelsolin-related proteins speaks for this problem.
Reply Yes, the the Triton-Insoluble Fraction (TIF) obtained from our extraction procedures includes cytoskeletal proteins. As described for your point 1, we have carefully changed the wording throughout the paper.

4- In fact, the relatively profound differences reported for lipids appear to me to be exaggerated for a short 30 minutes onlytreatment with polyP. Most of the lipids reported are not signalling molecules, but metabolic and/or structural lipids.
Reply We agree, it is indeed surprising that the detergent-resistant lipid/ cytoskeleton changes in 30 minutes. We did the detergent-resistant lipid/ cytoskeleton analysis after seeing that a 30-minute exposure of cells to polyphosphate changed the fluorescence response after photobleaching, and polyP affects membrane recycling with an even shorter timescale. With such effects on the bulk properties of the plasma membrane, one would expect to observe effects on cytoskeleton-associated structural lipids.

Minor comments:

1- The lipid species in Fig 5 "17-Pentatriacontene" is presented twice and with different results.
Reply We apologize for this error. We have updated the figure with the correct data.

2- The classification of proteins in Table 3 as lipid raft components is almost fanciful. In addition, lipids are not"up/downregulated", but maybe under/overabundant.
Reply The table 3 (now Table S2) and it's legend is updated now. Upregulated and downregulated are replaced by overbundant and underabundant, respectively. In line 278 of result section, we replaced "upregulated" with "increased the abundance of…".

3- In Figures 1F, G and S1B, please, use a linear X axis scale, otherwise the representation is distorted and misleading.
Reply We changed the X-axis scale to linear in Figure 1F, G and S1B.

4- Line 314, the argument about uncoupling of endocytosis and exocytosis appear weird. Cells would either shrink or inflate todeath!?
Reply We have updated the paragraph to "Although polyP partially inhibits exocytosis of the vesicles containing partially digested or undigested food, we do not know if polyP affects other secretory pathways involved in protein secretion that are necessary for cell- cell communication (Buratta, 2020). We previously observed that polyP inhibits cytokinesis to increase the percentage of large cells (Suess and Gomer, 2016), and it is possible that *D. discoideum* increases cell size while inhibiting exocytosis and membrane recycling using other mechanisms that add material to the plasma membrane." (lines 319- 325).

5- Line 327, autophagy is not (directly) linked to phagocytosis and therefore, not to killing of ingested bacteria.
Reply We have changed that sentence to "Low concentrations of polyP (500 nM) increase mTOR activity (Wang et al., 2003), and activated mTOR may inhibit autophagy, perhaps as a mechanism to inhibit the killing of ingested bacteria." (lines 333-335)

6- Figure 1D, it is hard to imagine that the technique to detect surface p80 works reliably and quantitatively, because it is knownthat PFA fixation partially damages and permeabilises the plasma membrane
Reply PFA will indeed cause partial damage to the membrane. We used permeabilized cells (top two rows in Figure 1D) as a control, and we consistently see that polyP reduces p80 surface staining without affecting the (control) total p80 staining.

© 2022. Published by The Company of Biologists under the terms of the Creative Commons Attribution License (https://creativecommons.org/licenses/by/4.0/).

7- In Fig 3, the FRAP is performed right in the center of the cell, likely affecting the whole cell volume, and not only the plasmamembrane.
Reply Yes, you are correct, cells were pretty sensitive to the high intensity laser that we used to photo-bleach the cells. In many instances, we have observed blebbing of the photo-bleached cells in the presence of polyphosphate, which might be due to increased rigidity of the membrane. However, intact cells were chosen to analyze the FRAP, and the cells were randomly chosen to photo-bleach at random locations on the cells. We have clarified this in the methods section on lines 505 to 507.

Reviewer 2 Advance Summary and Potential Significance to Field:
This paper makes a bold statement, that polyphosphate changes membrane fluidity and thus changes the nutritional state of cells acting through a signalling pathway. It this is true it's a very interesting story.

Reviewer 2 Comments for the Author:

1- The key point to differentiate is between polyphosphate just physically obstructing membrane traffic and thus the author's measure of fluidity, and between the receptor-mediated pathway they're describing. I'd be suspicious that the concentrations of polyphosphate they'd describe would obstruct vesicle traffic.The authors have tested this, explicitly, in Fig 3F-H. These figures are extremely important to justify the conclusions. But the statistics shown are wrong to support the point, and the results look worryingly ambiguous. If I understand right, 3C shows that half-time recovery is significantly different between control and 3 different polyphosphate preps. 3F does not show a significant difference when the same test is done on grlD- cells. But (a) the difference is very similar - it's just the error bars look worse in the control for grlD; (b) this is in any case not publishable stats. They need to test whether they can be confident that the ratio is different between grlD and wild type. Getting a significant experiment in one line and not one in the other doesn't mean anything; maybe the experiment on the mutant was less successful, for example. They need an explicit test (perhaps using ANOVA?) of difference. (c) the same is true for both of the other measures - it looks like polyP affects grlD- cells a lot in 3G and 3H. (d) the figure legend doesn't say whether the experiments in 3F-H were done with 'spectrum', 'filtered', 'short chain' or 'medium chain' preps; this completely changes the interpretation.The authors need to do a single experiment (in triplicate or more) comparing WT and grlD (etc) on the same day and the same conditions, then use a sound test to determine whether the effects of the treatment are different.
Reply We apologize for the confusion – we indeed did examine WT and grlD¯ on the same days. To save room, we put the WT data in 3C, and the mutant data in 3F. We have now copied the WT data from 3C into 3F. The same goes for the WT data from 3D now being added to 3G, and WT data from 3E now added to 3H. As above, the WT and mutant experiments were indeed done at the same time. The differences between no polyP and polyP treated grlD- cells in 3F-H are not significant. We have updated the figure legend in line 900-903 to "**(F-H)** Half-life of recovery, diffusion coefficient, and mobile fraction were calculated for WT, *grlD¯*, *ppk1¯*, *i6kA¯*, and *i6kA¯/i6kA* as in **B-E** using spectrum polyP. WT data with and without polyP in C to H are the same and all the experiments with mutants were done together with WT."

2- Another thing I'd question - the authors come to the conclusion that wild type cells are spitting out digestible food, based pretty exlusively on the data in Fig S1I. Those are interesting, but not nearly clear enough to justify the strong conclusions in the text and abstract. Maybe cells eat all their food (you'd think so, biologically) and cell killing reports something different based on membrane fluidity/recycling. I would try and strengthen this data; and in any case I would greatly tone down the conclusions about cells not digesting bacteria they've phagocytosed, perhaps to the discussion, not theresults and abstract.
Reply What Fig S1I is showing is that high concentrations of extracellular polyP inhibit the killing of a small percentage of ingested bacteria, in agreement with our previous observation that lower concentrations of polyP inhibit the killing of a small percentage of ingested bacteria without affecting ingestion (phagocytosis) (Rijal et al., 2020). We have carefully avoided any discussion of whether cells are spitting out digestible food,

© 2022. Published by The Company of Biologists under the terms of the Creative Commons Attribution License (https://creativecommons.org/licenses/by/4.0/).

everything in this manuscript points to the idea that when Dicty cells sense that they are at a high cell density, as indicated by a high extracellular concentration of polyP, they simply slow down excretion.

3- are they really measuring fluidity, not micropinocytosis/exocytosis? Please discuss more...
Reply We have updated the discussion on membrane fluidity by adding “Physical properties of cell membranes, such as membrane fluidity, are critical determinants of efficient endocytosis and exocytosis in mammalian cells (Ben-Dov and Korenstein, 2013; Ge et al., 2010).” (lines 301-303)

4- do you really want to conclude "PolyP alters the protein composition of lipid microdomains" from the data in Fig. 5? That looks like a max 4% difference. It may be stasticially significant, but can that small a difference have any biological relevance?
Reply Figure 5 is fatty acid esters, not proteins; the lipid raft proteins whose abundances in the detergent-insoluble fraction are altered by polyP exposure are in Table 3; total proteins in the detergent-insoluble fraction are in Supplementary Table 1. The data in Figure 5, along with the data in Tables 1 and 2, support the idea that polyP exposure does have an effect on some, but not all, lipids in the detergent-insoluble fraction. How the lipid and protein composition of the detergent-insoluble fraction affects biological processes in eukaryotic cells is very poorly understood, and we hope that this manuscript will be a contribution to this very complex field.

Second decision letter

MS ID#: JOCES/2022/260107

MS TITLE: Dictyostelium discoideum cells retain nutrients when the cells are about to overgrow their food source

AUTHORS: Ramesh Rijal, Sara Ann Kirolos, Ryan J Rahman, and Richard Gomer
ARTICLE TYPE: Research Article

We have now reached a decision on the above manuscript.

To see the reviewer's reports and a copy of this decision letter, please go to: https://submit-jcs.biologists.org and click on the 'Manuscripts with Decisions' queue in the Author Area. (Corresponding author only has access to reviews.)

As you will see, only one reviewer provided further comment. That reviewer has raise a number of substantial criticisms that prevent me from accepting the paper at this stage. Their critical point relates to analysis of the Triton Insoluble Floating Fraction to define the analysis of "rafts". I agree with them that this is a critical point (they suggest analysis by sucrose gradient centrifugation in the first review). A revised version would likely prove acceptable, if you can address their concerns. If you think that you can deal satisfactorily with the criticisms on revision, I would be pleased to see a revised manuscript. We would then return it to the reviewers.

Please ensure that you clearly highlight all changes made in the revised manuscript. Please avoid using 'Tracked changes' in Word files as these are lost in PDF conversion.

I should be grateful if you would also provide a point-by-point response detailing how you have dealt with the points raised by the reviewers in the 'Response to Reviewers' box. Please attend to all of the reviewers' comments. If you do not agree with any of their criticisms or suggestions please explain clearly why this is so.

© 2022. Published by The Company of Biologists under the terms of the Creative Commons Attribution License (https://creativecommons.org/licenses/by/4.0/).

Reviewer 1

Advance summary and potential significance to field

As highlighted in my original evaluation, I find the study timely and on an important topic. The results are interesting, though still slightly intriguing, but are well worth sharing with the community.

Comments for the author

Overall, the authors have performed a reasonable job to revise their manuscript, although most responses are in the form of argumentation, changes of wording, softening of claims, and did not result in any experimental modifications. Therefore, I will let it up to the Editor to decide whether these "cosmetic", somewhat superficial changes are enough to warrant publication as is.
In any case, four instances where the authors have misinterpreted my original major comments and did not bring a satisfying response.

1- I am well aware for decades that the protocols for "cytoskeleton preparations" and lipid rafts are very similar to say the least. Now, that was not my first point. As much as I know, the use of TritonX100 (and for that matter of any other detergent) normally precludes a lipidomic analysis by MS, or at least "messes it up". Therefore, how reliable is the MS analysis, technically?

2- I accept the arguments about the silica bead contaminant and the question mark on cholestanol ... even though, to my knowledge it derives only from the catabolism of cholesterol ... which is NOT produced by Dictyostelium Is that another contaminant? But the major point is about TAGs and DAGs, which are NOT membrane components (DAG can be produced in very small amounts in membranes) and even with the new broader definition, are NOT cytoskeleton components. TAGs are found exclusively in lipid droplets which should NOT be in the TIF pellet. Howe reliable is the method and how contaminated are these lipid fractions?

3- It is true that the TIF pellet corresponds to cytoskeletal proteins and DRMs ... but not rafts! The authors ignore my point, which is that rafts are in TIFFs, the Triton Insoluble FLOATING fraction. Therefore, any link to domains, membrane composition and biophysical characteristics can only be linked to TIFFs. Please perform this experiment/analysis.

Minor point 7- The authors miss the point. I am aware that even if done perfectly appropriately, the laser used for FRAP induces large scale injuries and morphology changes. This is in fact reasonable here, as seen in the movies. But my point is that people usually target the periphery of the cell, in order to target majorly the plasma membrane. Here, the laser is shot in the nuclear area, which beaches (and damages) a large voxel/volume of the cytoplasm, contains organelles etc... and not only the plasma membrane. Please perform your FRAP at the periphery or rephrase/soften your conclusions.

Second revision

Author response to reviewers' comments

Reviewer 1 Advance summary and potential significance to field
As highlighted in my original evaluation, I find the study timely and on an important topic. The results are interesting, though still slightly intriguing, but are well worth sharing with the community.
Reply- Thank you. We truly appreciate your effort to make this manuscript clear.

Reviewer 1 Comments for the author

Overall, the authors have performed a reasonable job to revise their manuscript, although most responses are in the form of argumentation, changes of wording, softening of claims, and did not

© 2022. Published by The Company of Biologists under the terms of the Creative Commons Attribution License (https://creativecommons.org/licenses/by/4.0/).

result in any experimental modifications. Therefore, I will let it up to the Editor to decide whether these "cosmetic", somewhat superficial changes are enough to warrant publication as is.
In any case, four instances where the authors have misinterpreted my original major comments and did not bring a satisfying response.
1- I am well aware for decades that the protocols for "cytoskeleton preparations" and lipid rafts are very similar to say the least. Now, that was not my first point. As much as I know, the use of TritonX100 (and for that matter of any other detergent) normally precludes a lipidomic analysis by MS, or at least "messes it up". Therefore, how reliable is the MS analysis, technically?
Reply- The lipidomics work was a very minor and ancillary part of this report. Since the standard method used to identify detergent resistant lipids involves a detergent extraction (almost always Triton X-100), and we can think of no possible way to determine whether or not the protocol would “mess up” the lipidomics, we have removed our lipidomic analysis data from the manuscript, and we now have removed Figure 5 and Tables 1 and 2 and the associated Methods and Results section. All changes are highlighted in yellow throughout the manuscript.

2- I accept the arguments about the silica bead contaminant and the question mark on cholestanol ... even though, to my knowledge it derives only from the catabolism of cholesterol ... which is NOT produced by Dictyostelium Is that another contaminant? But the major point is about TAGs and DAGs, which are NOT membrane components (DAG can be produced in very small amounts in membranes) and even with the new broader definition, are NOT cytoskeleton components. TAGs are found exclusively in lipid droplets, which should NOT be in the TIF pellet. Howe reliable is the method and how contaminated are these lipid fractions?
Reply- As described above, we have removed all of the lipidomics work.

3- It is true that the TIF pellet corresponds to cytoskeletal proteins and DRMs ... but not rafts! The authors ignore my point, which is that rafts are in TIFFs, the Triton Insoluble FLOATING fraction. Therefore, any link to domains, membrane composition and biophysical characteristics can only be linked to TIFFs. Please, perform this experiment/analysis.
Reply- We have found some known lipid raft proteins in the crude cytoskeletal extract (SUPPLEMENTARY TABLE 2), so polyP might be altering membrane cytoskeletal proteins that are also components of the lipid rafts, and several papers discuss the presence of rafts in DRMs. In addition, we are aware that the biophysical membrane properties are not only linked to TIFFs, but also to TIFs. Since the reviewer has raised concerns about the use of detergent extractions for lipidomics, we do not think that further experiments are warranted, since any examination of TIFFs would look really odd with no lipidomics. We have clarified the text to make sure that we are not claiming that the crude cytoskeletal extract preparations are exclusively rafts or microdomains, and added relevant references to the text on page 12.
Minor point 7- The authors miss the point. I am aware that even if done perfectly appropriately, the laser used for FRAP induces large scale injuries and morphology changes. This is in fact reasonable here, as seen in the movies. But my point is that people usually target the periphery of the cell, in order to target majorly the plasma membrane. Here, the laser is shot in the nuclear area, which beaches (and damages) a large voxel/volume of the cytoplasm, contains organelles etc... and not only the plasma membrane. Please, perform your FRAP at the periphery or rephrase/soften your conclusions.
Reply- We followed the protocol from the published article (Tanaka et al., 2017) to perform the FRAP analysis. This protocol involves targeting the main body of the cell. The only difference was that the Tanaka paper used a 5 µm diameter spot, and to reduce damage to the cells, we used a 3 µm diameter spot. We are aware of the damage the laser could do to the cells. We tried targeting the cell edges to perform FRAP, but we were unsuccessful because of the rapid movement of the edge of the cells, as Dicty cells are highly motile. It is indeed possible that the laser could damage the cells, and the fluorescence recovery we have observed might be the consequence of that injury. We have rephrased and softened our conclusion on page 9.

© 2022. Published by The Company of Biologists under the terms of the Creative Commons Attribution License (https://creativecommons.org/licenses/by/4.0/).

Third decision letter

MS ID#: JOCES/2022/260107

MS TITLE: Dictyostelium discoideum cells retain nutrients when the cells are about to overgrow their food source

AUTHORS: Ramesh Rijal, Sara Ann Kirolos, Ryan J Rahman, and Richard Gomer
ARTICLE TYPE: Research Article

I am happy to tell you that your manuscript has been accepted for publication in Journal of Cell Science, pending standard ethics checks. Thank you for making those additional modifications to the manuscript. I agree that removing the lipidomics simplifies the manuscript. There are likely to be some copyediting queries which I encourage you to look out for around the use of cytoskeletal/cytoskeleton.

© 2022. Published by The Company of Biologists under the terms of the Creative Commons Attribution License (https://creativecommons.org/licenses/by/4.0/).
